# Adaptive two-stage inverse sampling design to estimate density, abundance, and occupancy of rare and clustered populations

**DOI:** 10.1371/journal.pone.0255256

**Published:** 2021-08-18

**Authors:** Mohammad Salehi, David R. Smith

**Affiliations:** 1 Department of Mathematics, Statistics and Physics, Qatar University, Doha, Qatar; 2 U.S. Geological Survey, Eastern Ecological Science Center, Kearneysville, WV, United States of America; Universidad Rey Juan Carlos, SPAIN

## Abstract

Sampling rare and clustered populations is challenging because of the effort required to find rare units. Heuristically, a practitioner would prefer to discontinue sampling in areas where rare units of interest are apparently extremely sparse or absent. We take advantage of the characteristics of inverse sampling to adaptively inform practitioners when it is efficient to move on to sample new areas. We introduce Adaptive Two-stage Inverse Sampling (ATIS), which is designed to leave a selected area after observation of an a priori number of only non-rare units and to continue sampling in the area when rare units are observed. ATIS is efficient in many cases and yields more rare units than conventional sampling for a rare and clustered population. We derive unbiased estimators of population total and variance. We also introduce an easy-to-compute estimator, which is nearly as efficient as the unbiased estimator. A simulation study on a rare plant population of buttercups (*Ranunculus*) shows that ATIS even with the easy-to-compute estimator is more efficient than its conventional sampling counterparts and is more efficient than Two-stage Adaptive Cluster Sampling (TACS) for small and moderate final sample sizes. Additional simulations reveal that ATIS is efficient for binary data (e.g., presence or absence) whereas TACS is inefficient for binary data. The overall results indicate that ATIS is consistently efficient compared to conventional sampling and to adaptive cluster sampling in some important cases.

## Introduction

Inverse sampling is adaptive in the sense that the total sampling effort depends on the stochastic observation of units that meet a specified characteristic. Units are selected following inverse sampling procedure until a predetermined number that meet the specified characteristic have been observed [1]. Haldane [2] used inverse sampling to estimate the frequency of a rare disease leading to other applications of inverse sampling to study rare populations.

Inverse sampling is slightly inefficient, in the sense of having smaller variance, than a Simple Random Sampling without replacement (SRS) with equal effective or expected final sample sizes. However, inverse sampling finds slightly more rare events than a SRS with equal sample sizes.

Salehi and Seber [3] showed that Murthy’s estimator [4] is appropriate for developing estimators for sequential sampling such as inverse sampling. Following [3], there has been quite a number inverse sampling design estimators developed using Murthy’s estimator. Moradi et al. [5] developed regression estimator under inverse sampling to estimate arsenic contamination. Aggarwaland and Pandey [6] used inverse sampling to study disease burden of leprosy in an endemic area of Uttar Pradesh, India. Salehi et al. [7] introduced inverse adaptive cluster sampling with unequal selection probabilities to study crab holes. Panahbehagh and Smith [8] developed group inverse sampling which is practical for field implementation. Mohammadi [9] has developed a bootstrap confidence intervals for inverse sampling. Latpate and Kshirsagar [10] introduced two-stage inverse adaptive cluster sampling with a stopping rule that depends on cluster size to control the final sample size.

In the literature, whereas authors developed Murthy’s estimators to study rare and clustered populations, the relative complexity their estimators deterred practitioners use, we believe. In response, Panahbehagh [11] recently proposed a resampling method to compute Murthy’s estimator to lower the computational barriers for practitioners.

Inverse sampling is generally perceived to continue until an a priori fixed number of rare units are observed. However, having a predetermined number of rare units is not our primary objective in this research. We develop a sampling design based on a heuristic that one should leave an area when no rare units are observed in an initial sample of the area and continue sampling the area when some rare units are observed initially.

To setup the design, let the population be partitioned into Primary Sampling Units (PSUs) to constrain sampling within areas. We select some of the PSUs in the first stage, and we then select an initial sample of secondary units from each of the selected PSUs. If we do not find rare units among the initial sample, we leave the PSU. If we find some rare units in the PSU, we will keep sampling one unit at a time, sequentially, until we observe the same number of non-rare units as in the initial sample size. Because we keep sampling until observing a predetermined number of non-rare units, the design is a form of “reverse-inverse” sampling.

For its sake of simplicity, we call the design Adaptive Two-stage Inverse Sampling (ATIS). We use Murthy’s estimator to analytically develop its variance estimator. We also develop an easy-to-compute estimator and its variance estimator, which is almost as efficient as the Murthy’s estimator. We believe that the estimator’s simplicity, along with the design’s efficiency and yield of rare units, will be attractive to practitioners.

During the last two decade, several adaptive sampling designs were introduced to sample rare and clustered populations, for example, two-stage sequential sampling [12], adaptive web sampling [13], and complete allocation sampling [14]. However, Adaptive Cluster Sampling (ACS) introduced by Thompson [15] and its different versions, stratified ACS [16], two-stage ACS [17] are still the foundation for sampling rare and clustered populations. Two-stage Adaptive cluster Sampling (TACS) and ATIS can be considered as competing options. Using a simulation study on the buttercups (*Ranunculus*) population, we show that ATIS is more efficient than TACS for small and moderate sample sizes but TACS is more efficient for large sample sizes. Site occupancy rate (the proportion of units occupied by a species) is critical information for many large scale and long-term conservation efforts for imperilled species [18, 19]. ACS and its different versions are inefficient sampling designs to estimate occupancy rate where the variable of interest is binary. Using a simulation study on a population, we show that ATIS is an efficient sampling design to estimate occupancy rate for rare and clustered populations characteristic of imperilled species. The standard recommendation is to use a modelling approach for estimation of occupancy to account for imperfect detection [20]. Pacifici et al. [19] integrated adaptive cluster sampling into occupancy modelling for spatially-clustered populations. However, Welsh et al. [21] found that when data are sparse, as is expected for rare species, occupancy modelling can perform poorly with errors commensurate with disregarding detectability. The potential gains in efficiency from adaptive designs are eroded by low detectability [22]. Plug-in estimators are available to incorporate independently estimated detectability in adaptive designs [23], but garnering independent estimates of detectability for rare species are not commonly available due to sparse data. Thus, we proceed with the assumption that detectability is high (>0.8; [22]) within a sampling unit so that efficiency and yield are the overriding concerns.

In summary, ATIS, which mimics how practitioners (e.g., conservation biologists) would like to collect data, has an easy-to-compute estimator, is a competitive option TACS to estimate parameters of a rare and clustered population, and is efficient for binary variables. Moreover, ATIS is a neighborhood-free sampling design which is an advantage over TACS design. If an appropriate neighborhood definition is not implemented, ACS and its TACS version will fail to detect the rare clusters as is demonstrated in section 3 (cf. [24, 25]).

In section 2, we develop a unbiased estimator and its variance estimator of the introduced sampling design based on Murthy’s estimator. To simplify the estimator, we then ignore the last selected non-rare unit in those PSUs for which we have sequentially selected extra units. By ignoring the last selected units, its estimator becomes as simple as the conventional two-stage simple random sample estimator. In section 3, ATIS properties will be studied. Using simulation studies, we compare ATIS with SRS, conventional two-stage sampling (CTS) and TACS. We then conclude the paper in section 4 by summarizing the results and providing some recommendations.

## Sampling design and terminology

### Sampling design

Suppose that we have a population of *N* units, which are partitioned into *M* primary units of size *N*_*i*_, (*i* = 1, 2, …, *M*), secondary units. Ideally, the primary units dimensions would be based on available information about the spatial distribution and size of clusters using prior survey information, habitat maps, or satellite images. Let unit (*i*, *j*) denote the *j*th secondary unit in the *i*th primary unit with an associated measurement or the count of a species of interest of *y*_*ij*_. Let $\tau_{i}=\sum_{j=1}^{N_{i}}y_{ij}$ be the sum of *y*-values in the *i*th primary unit, and let $\tau =\sum_{i=1}^{M}\tau_{i}$ be the population total. The population of secondary units in primary unit *i* is divided into two subpopulations according to whether the *y*-values satisfy a condition *C*, for example *C* = {*y*_*ij*_: *y*_*ij*_ > *c*}, where *c* is a constant. Let denote the two subpopulations by $P_{iC}=\left\{u:y_{ij}\in C,j=1,\ldots ,N_{i}\right\}$ and $P_{iC^{\prime }}=\left\{u:y_{ij}\notin C,j=1,\ldots ,N_{i}\right\}$, where $K_{i}=\left|P_{iC^{\prime }}\right|$ and $N_{i}-K_{i}=\left|P_{iC}\right|$ are the unknown numbers of units, or cardinalities, of $P_{iC^{\prime }}$ and $P_{iC}$, respectively. In the first stage, we choose a sample of size *m* from the *M* primary units in the population using a sampling design with inclusion probability *π*_*i*_ of primary unit *i* and the joint inclusion probability *π*_*ii*′_ of primary units *i* and *i*′. In the second stage, we select a simple random sample of size *k*_*i*_ secondary units without replacement from primary unit *i*, *i* = 1, 2, …, *m*. If all observed units are from $P_{iC^{\prime }}$ there will be no further sampling in primary unit *i*. If the initial sample contains less than *k*_*i*_ units from $P_{iC^{\prime }}$ sampling continues in a sequential manner, one at a time, until exactly *k*_*i*_ units are selected from $P_{iC^{\prime }}$. In other words, *k*_*i*_ is a threshold which is used as a rule of thumb to leave primary units *i*. Let *ν*_*i*_ be the final sample size from PSU *i*.

**Fig 1:**
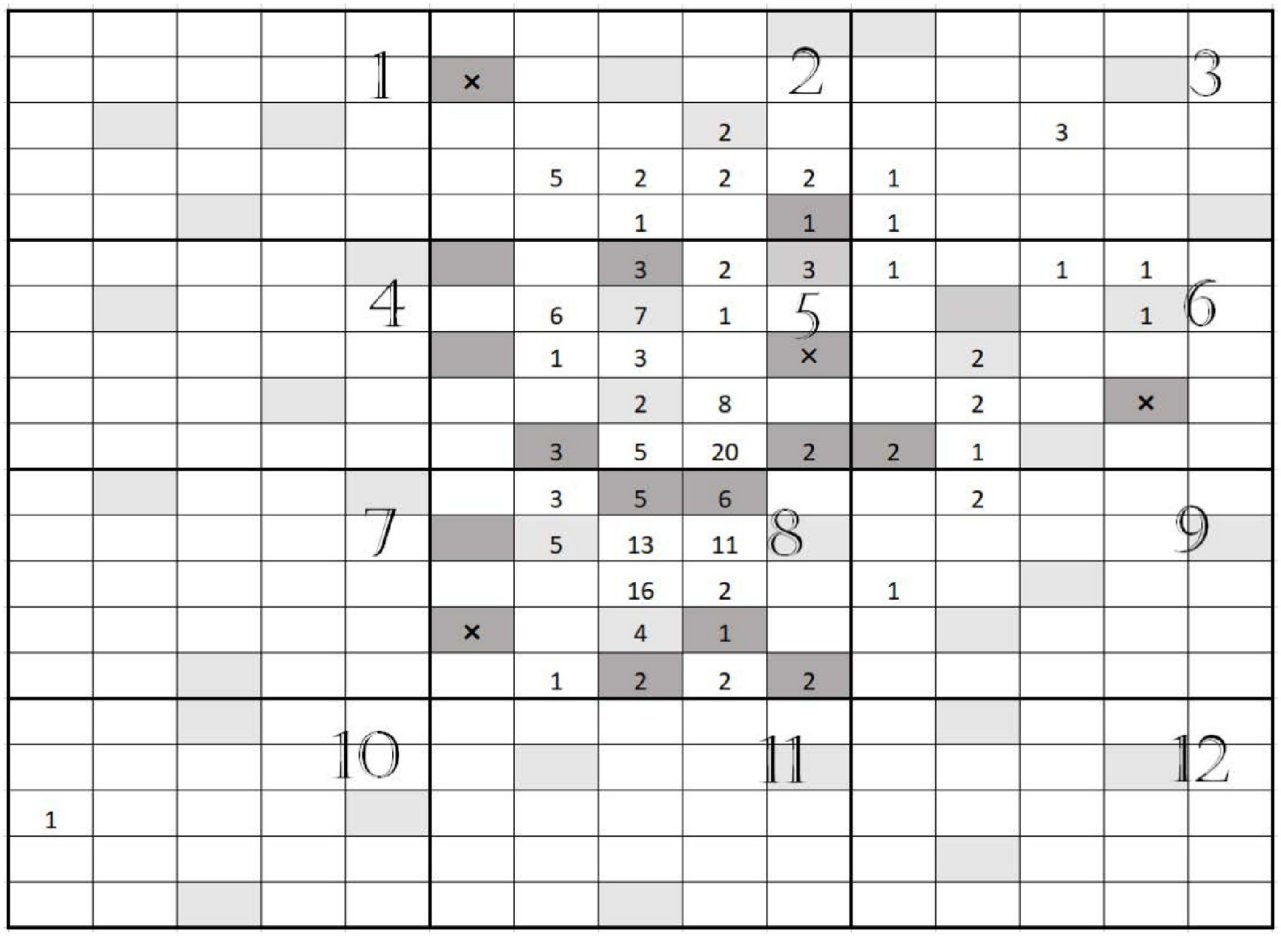
Castle Hill buttercups population. There are 300 quadrats of size 100*m*^2^. The counts of buttercups are shown. The population site is partitioned into 12 primary units. All 12 primary units are selected. Three quadrats are selected from each PSU, in light gray. If all three selected quadrates are not empty, the sampling has been continued one at a time, sequentially, until three empty quadrats are selected. The sequentially selected quadrates are in dark gray and the last selected quadrats have × which are non-rare quadrates.

To illustrate, PUS 8 in Fig 1 shows a PSU of size 25 with 14 rare units, which have a number in them. A SRS of size 3 is selected with units in light gray. Two selected units are rare units and the other unit is non-rare. The sampling procedure continues one at a time until two more non-rare units is selected. The extra selected units are in dark gray which are 7 units from which 5 are rare units. The final sample contains 7 rare units and 3 non-rare units. The last selected units is the one with ×.

### Estimator and its variance estimator

Salehi and Seber [3] showed that Murthy’s estimator can provide an unbiased estimator for sequential sampling designs. Murthy’s estimator is

$$\begin{array}{c} {\hat{\tau }}_{i}=\sum_{j\in s_{i}}\frac{P(s_{i}|j)}{P(s_{i})}y_{ij} \end{array}$$

where *P*(*s*_*i*_) is the probability of finally obtaining the sample set *s*_*i*_ in primary unit *i* and *P*(*s*_*i*_|*j*) is the conditional probability of getting the sample *s*_*i*_ given the *j*th unit was selected in the first draw in primary unit *i*. The variance of ${\hat{\tau }}_{i}$ is given by

$$\begin{array}{c} \text{var}\left[{\hat{\tau }}_{i}\right]=\sum_{j=1}^{N_{i}}\sum_{j<j^{\prime }}^{N_{i}}(1-\sum_{s_{i}∍j,j^{\prime }}\frac{P\left(s_{i}|j\right)P\left(s_{i}|j^{\prime }\right)}{P(s_{i})}){\left(\frac{y_{ij}}{p_{j,i}}-\frac{y_{ij^{\prime }}}{p_{j^{\prime },i}}\right)}^{2}p_{j,i}p_{j^{\prime },i}, \end{array}$$

where *p*_*j*,*i*_ the probability that unit *j* in primary unit *i* is selected first at the second stage. Because we have *p*_*j*,*i*_ = 1/*N*_*i*_ for all *j* = 1, 2, …, *N*_*i*_,

$$\begin{array}{c} \text{var}\left[{\hat{\tau }}_{i}\right]=\sum_{j=1}^{N_{i}}\sum_{j<j^{\prime }}^{N_{i}}(1-\sum_{s_{i}∍j,j^{\prime }}\frac{P\left(s_{i}|j\right)P\left(s_{i}|j^{\prime }\right)}{P(s_{i})}){\left(y_{ij}-y_{ij^{\prime }}\right)}^{2}, \end{array}$$

and its unbiased estimator is

$$\begin{array}{c} \hat{\text{var}}\left[{\hat{\tau }}_{i}\right]=\sum_{j\in s_{i}}\sum_{j<j^{\prime }}\left(\frac{P(s_{i}|jj^{\prime })}{P(s_{i})}-\frac{P\left(s_{i}|j\right)P\left(s_{i}|j^{\prime }\right)}{P{\left(s_{i}\right)}^{2}}\right){\left(y_{ij}-y_{ij^{\prime }}\right)}^{2}, \end{array}$$

where *P*(*s*_*i*_|*jj*′) is the probability of the sample *s*_*i*_ given that the units *j* and *j*′ were selected regardless of order in the first two draws in primary unit *i*.

Using the definition of conditional probability and a simple algebra we have

$$\begin{array}{c} {\hat{\tau }}_{i}=\sum_{j\in s_{i}}\frac{P(j|s_{i})}{p_{j,i}}y_{ij} \end{array}$$ (1)

and its variance estimator of (1) is

$$\begin{array}{c} \hat{\text{var}}\left[{\hat{\tau }}_{i}\right]=\sum_{j\in s_{i}}\sum_{j<j^{\prime }}\left(\frac{P(jj^{\prime }|s_{i})}{p_{j,i}}-\frac{P\left(j|s_{i}\right)P\left(j^{\prime }|s_{i}\right)}{p_{j,i}p_{j^{\prime },i}}\right){\left(y_{ij}-y_{ij^{\prime }}\right)}^{2}, \end{array}$$ (2)

Evaluating (1) and (2) for ATIS design, we have

$$\begin{array}{c} {\hat{\tau }}_{i}=N_{i}({\hat{P}}_{i}{\bar{y}}_{iC^{\prime }}+\left(1-{\hat{P}}_{i}\right){\bar{y}}_{iC}), \end{array}$$ (3)

where ${\hat{P}}_{i}=\left(k_{i}-1\right)/\left(\nu_{i}-1\right)$, ${\bar{y}}_{iC^{\prime }}=k_{i}^{-1}\sum_{j\in S_{iC^{\prime }}}y_{ij}$, ${\bar{y}}_{iC}={\left(\nu_{i}-k_{i}\right)}^{-1}\sum_{i\in S_{iC}}y_{ij}$ and, $S_{iC}$ are $S_{iC^{\prime }}$ are samples from $P_{iC}$ and $P_{iC^{\prime }}$, respectively. The derivation is given in S1 Appendix. Note that if all *k*_*i*_ selected units do not satisfy *C*, ${\hat{P}}_{i}$ is 1 so that ${\hat{\tau }}_{i}=N_{i}{\bar{y}}_{iC^{\prime }}$. An unbiased estimator of the variance of (3) is given by

$$\begin{array}{c} \hat{\text{var}}\left({\hat{\tau }}_{i}\right)=\{\begin{array}{cc} N_{i}^{2}\left(\frac{1}{\nu_{i}}-\frac{1}{N_{i}}\right)s_{iC^{\prime }}^{2} & \nu_{i}=k_{i}\\ N_{i}^{2}(As_{iC^{\prime }}^{2}+\hat{\text{var}}\left({\hat{P}}_{i}\right){\left({\bar{y}}_{iC^{\prime }}-{\bar{y}}_{iC}\right)}^{2}+Bs_{iC}^{2}) & \nu_{i}>k_{i}, \end{array} \end{array}$$ (4)

where

$$\begin{array}{c} \hat{\text{var}}\left({\hat{P}}_{i}\right)=\left(1-\frac{\nu_{i}-1}{N_{i}}\right)\frac{{\hat{P}}_{i}\left(1-{\hat{P}}_{i}\right)}{\nu_{i}-2}, \end{array}$$

$$\begin{array}{c} A=\hat{\frac{P_{i}^{2}}{k_{i}}}(\frac{\left(N_{i}-\nu_{i}+1\right)\left(\nu_{i}k_{i}-\nu_{i}-k_{i}\right)-N_{i}\left(\nu_{i}-2\right)}{N_{i}\left(\nu_{i}-2\right)\left(k_{i}-1\right)}),B=\frac{\left(N_{i}-\nu_{i}+1\right)\left(\nu_{i}-k_{i}-1\right)}{N_{i}\left(\nu_{i}-1\right)\left(\nu_{i}-2\right)}, \end{array}$$

$$\begin{array}{c} s_{iC^{\prime }}^{2}=\frac{1}{k_{i}-1}\sum_{j\in S_{iC^{\prime }}}{\left(y_{ij}-{\bar{y}}_{iC^{\prime }}\right)}^{2}\;\text{and}\;s_{iC}^{2}=\frac{1}{\nu_{i}-k_{i}-1}\sum_{j\in S_{iC}}{\left(y_{ij}-{\bar{y}}_{iC}\right)}^{2} \end{array}$$

The estimate of the total population, *τ*, is

$$\begin{array}{c} \hat{\tau }=\sum_{i=1}^{m}\frac{{\hat{\tau }}_{i}}{\pi_{i}}, \end{array}$$

where *π*_*i*_ is the inclusion probability PSU *i* ([26], p. 89), and an unbiased estimator of its variance is

$$\begin{array}{c} \hat{\text{var}}\left(\hat{\tau }\right)=\sum_{i=1}^{m}\sum_{i^{\prime }=1}^{m}(\frac{1}{\pi_{i}\pi_{i^{\prime }}}-\frac{1}{\pi_{ii^{\prime }}}){\hat{\tau }}_{i}{\hat{\tau }}_{i^{\prime }}+\sum_{i=1}^{m}\frac{\hat{\text{var}}\left({\hat{\tau }}_{i}\right)}{\pi_{i}}, \end{array}$$

where *π*_*ii*_ is the joint inclusion probability and *π*_*ii*_ = *π*_*i*_.

In practice, if the sizes of primary units are the same and auxiliary variables are not available, SRS design would be a reasonable choice for the first stage (Salehi and Smith 2005). If the first stage design is SRS, then an unbiased estimator is

$$\begin{array}{c} \hat{\tau }=M\bar{\hat{\tau }}=\frac{M}{m}\sum_{i=1}^{m}N_{i}({\hat{P}}_{i}{\bar{y}}_{iC^{\prime }}+\left(1-{\hat{P}}_{i}\right){\bar{y}}_{iC}), \end{array}$$ (5)

where $\bar{\hat{\tau }}=\sum_{i=1}^{m}{\hat{\tau }}_{i}/m$. An unbiased estimator of its variance is

$$\begin{array}{c} \hat{\text{var}}\left(\hat{\tau }\right)=M\left(M-m\right)\frac{s_{\tau }^{2}}{m}+\frac{M}{m}\sum_{i=1}^{m}\hat{\text{var}}\left({\hat{\tau }}_{i}\right), \end{array}$$ (6)

where $s_{\tau }^{2}=\sum_{i=1}^{m}{\left({\hat{\tau }}_{i}-\bar{\hat{\tau }}\right)}^{2}/\left(m-1\right).$

#### An easy-to-compute estimator and its variance estimator

Pathak [27] introduced an unbiased estimator for the mean population in fixed cost sequential sampling schemes. The estimator is the sample mean where the last selected unit is ignored. Using Pathak’s approach, we may show that

$$\begin{array}{c} {\tilde{\tau }}_{i}=\{\begin{array}{cc} N_{i}\left[\frac{1}{\nu_{i}}\sum_{j=1}^{\nu_{i}}y_{ij}\right]=N_{i}{\bar{y}}_{\nu_{i}} & \nu_{i}=k_{i}\\ N_{i}\left[\frac{1}{\nu_{i}-1}\sum_{j=1}^{\nu_{i}-1}y_{ij}\right]=N_{i}{\bar{y}}_{\nu_{i}-1} & \nu_{i}>k_{i} \end{array} \end{array}$$

is an unbiased estimator, where ${\bar{y}}_{\nu_{i}-1}$ is the sample mean based on the first *ν*_*i*_ − 1 selected units. This estimator is inadmissable as the last selected sample is discarded. An estimator is inadmissible if it is uniformly dominated by some other estimator. Since $\text{var}({\hat{\tau }}_{i})$ is uniformly smaller than $\text{var}({\tilde{\tau }}_{i})$, ${\tilde{\tau }}_{i}$ is an inadmissable estimator. It can be showed that ${\hat{\tau }}_{i}$ is the Rao-Blackwell version of ${\tilde{\tau }}_{i}$. The ATIS is designed so that the last observed unit in PSUs for which the extra sequentially are selected, are non-rare so that the loss of information will be minimal. When *c* is zero in condition *C* = {*y*_*ij*_: *y*_*ij*_ > *c*} the easy-to-compute estimator ${\tilde{\tau }}_{i}$ will be equal to ${\hat{\tau }}_{i}$ for *i* = 1, …, *m* so that there is no loss of information. When the first stage design is a SRS, another unbiased estimator is,

$$\begin{array}{c} \tilde{\tau }=M\bar{\tilde{\tau }}, \end{array}$$ (7)

where $\bar{\tilde{\tau }}=\sum_{i=1}^{m}{\tilde{\tau }}_{i}/m$. This estimator can be easily computed and as simple as a Conventional Two-Stage (CTS) estimator. An unbiased estimator of its variance is

$$\begin{array}{c} \hat{\text{var}}\left(\tilde{\tau }\right)=M\left(M-m\right)\frac{s_{\tau }^{2}}{m}+\frac{M}{m}\sum_{i=1}^{m}\hat{\text{var}}\left({\tilde{\tau }}_{i}\right), \end{array}$$ (8)

where $s_{\tau }^{2}=\sum_{i=1}^{m}{\left({\tilde{\tau }}_{i}-\bar{\tilde{\tau }}\right)}^{2}/\left(m-1\right)$, and

$$\begin{array}{c} \hat{\text{var}}\left({\tilde{\tau }}_{i}\right)=\{\begin{array}{cc} N_{i}^{2}\left(\frac{1}{\nu_{i}}-\frac{1}{N_{i}}\right)s_{iC^{\prime }}^{2} & \nu_{i}=k_{i}\\ N_{i}^{2}\left(\frac{1}{\nu_{i}-1}-\frac{1}{N_{i}}\right)s_{\nu_{i}-1}^{2} & \nu_{i}>k_{i}, \end{array} \end{array}$$ (9)

where $s_{\nu_{i}-1}^{2}=\left(1/\left(\nu_{i}-2\right)\right)\sum_{j=1}^{\nu_{i}-1}{\left(y_{ij}-{\bar{y}}_{\nu_{i}-1}\right)}^{2}$, the sample variance of the first *ν*_*i*_ − 1 selected units in the PSUs with at least one rare unit observed.

The number of observed rare units in each primary unit has Negative Hypergeometric distribution [28, 29]. The expected number of observed rare units in each primary sampling unit is *k*_*i*_ *K*_*i*_/(*N*_*i*_ − *K*_*i*_+1). When the first stage sampling is simple random sample and $k_{i}^{\prime }\text{s}$ are the same, the expected final sample size is

$$\begin{array}{c} E\left(\nu \right)=\frac{m}{M}\sum_{1=i}^{m}E\left(\nu_{i}\right)=\frac{mk}{M}\sum_{i=1}^{M}\frac{N_{i}+1}{N_{i}-K_{i}+1}, \end{array}$$

and the expected number of observed rare units, say *ν*_*r*_, will be

$$\begin{array}{c} E_{ATIS}\left(\nu_{r}\right)=E\left(\nu -mk\right)=\frac{mk}{M}\sum_{i=1}^{M}\frac{K_{i}}{N_{i}-K_{i}+1}, \end{array}$$ (10)

where E*_ATIS_* is the expected value for ATIS design. We may compare the expected number of observed rare units, with that of a SRS design of size E(*ν*) which will be,

$$\begin{array}{c} E_{SRS}\left(\nu_{r}\right)=\left(\frac{mk}{M}\right)\left(\frac{K_{t}}{N}\right)\sum_{i=1}^{M}\frac{N_{i}+1}{N_{i}-K_{i}+1} \end{array}$$ (11)

where $K_{t}=\sum_{i=1}^{M}K_{i}$, the total rare units in the population. A fairer comparison is to compare (10) with the expected number of observed rare units for a conventional two-sage sampling of size *m* PSUs and of size E(*ν*)/*m* units in the selected PSUs which will be

$$\begin{array}{c} E_{CTS}\left(\nu_{r}\right)=\left(\frac{k}{M}\right)\left(\frac{m}{M}\right)\sum_{i=1}^{M}\frac{N_{i}+1}{N_{i}-K_{i}+1}\sum_{i=1}^{M}\frac{K_{i}}{N_{i}}, \end{array}$$ (12)

where E_*CTS*_ is the expected value for CTS design. We will use these formulas in the next section to compare the observed rare units of ATIS design with its counterparts.

## Study of ATIS properties

### An example

To shed light on computation, we used data are from a study on Castle Hill buttercups found within the Lance McCaskill Nature Reserve in the South Island of New Zealand [30]. The Castle Hill buttercup is one of New Zealand’s rarest plants [31]. Locations of buttercup plants observed were mapped within a 3 hectare area using 300 10 by 10 *m*^2^ plots (Fig 1).

To illustrate, the population was partitioned into 12 PSUs each 2,500 *m*^2^ in size. All PSUs were selected, *m* = *M*, in the first stage. (The PSU number is given on top-left of each PSU within Fig 1). Setting the condition to adapt at *y*_*ij*_ > 0, *k*_*i*_ = 3 and *m* = 12, three plots (secondary units) were selected from each PSU, the gray light plots. Some buttercups were found in PSUs 2, 5, 6 and 8. We therefore continue to select plots one at a time until we have 3 non-rare plots in those PSUs so that dark gray plots are selected. Plots with × indicate selection. The population total estimators ${\hat{\tau }}_{i}={\tilde{\tau }}_{i}=0$, for *i* = 1, 3, 4, 7, 9, 10, 11, 12. For *i* = 5, the final sample size is 9, *ν*_*i*_ = 9, and we have,

$$\begin{array}{c} {\tilde{\tau }}_{5}=25\left(\frac{0+0+3+3+7+2+3+2}{8}\right)=25\left(2.5\right)=62.5 \end{array}$$

$$\begin{array}{c} {\hat{\tau }}_{5}=25\left[\left(\frac{2}{8}\right)0+\left(\frac{6}{8}\right)\left(\frac{3+3+7+2+3+2}{6}\right)\right]=62.5 \end{array}$$

For other PSUs ${\tilde{\tau }}_{2}={\hat{\tau }}_{2}=18.75,\;{\tilde{\tau }}_{6}={\hat{\tau }}_{6}=25$ and ${\tilde{\tau }}_{8}={\hat{\tau }}_{8}=75.$ Thus,

$$\begin{array}{c} \tilde{\tau }=\hat{\tau }=181.25. \end{array}$$

To compute variance estimator of $\tilde{\tau }$, we use (8) for which the first term is zero as all PSUs are selected in the first stage, and the second term reduces to $\sum_{i=1}^{M}\hat{\text{var}}\left({\tilde{\tau }}_{i}\right)$. Substituting (9) into (8), we have

$$\begin{array}{c} \hat{\text{var}}\left(\tilde{\tau }\right)=\sum_{i=1}^{M}N_{i}^{2}\left(\frac{1}{\nu_{i}-1}-\frac{1}{N_{i}}\right)s_{\nu_{i}-1}^{2}. \end{array}$$

In S2 Appendix, we prove that $\hat{\text{var}}\left(\hat{\tau }\right)$ is exactly the same as $\hat{\text{var}}\left(\tilde{\tau }\right)$ when *c* is zero. For *i* = 1, 3, 4, 7, 9, 10, 11, 12, $s_{\nu_{i}}^{2}$ is zero; $s_{\nu_{5}-1}^{2}=0.1925$, $s_{\nu_{6}-1}^{2}=0.4128$ and $s_{\nu_{8}-1}^{2}=0.16$. We therefore have

$$\begin{array}{c} \hat{\text{var}}\left(\hat{\tau }\right)=\hat{\text{var}}\left(\tilde{\tau }\right)=709.21. \end{array}$$

### The expected number of observed rare units

For the buttercup population, Fig 1, the size of PSUs are the same so that $N_{i}=N/M=\bar{N}$ for *i* = 1, 2, …, *M*. Therefore, the expected number of observed rare units for a conventional two-sage sampling will be

$$\begin{array}{ccc} E_{CTS}\left(\nu_{r}\right) & = & \left(\frac{k}{M}\right)\sum_{i=1}^{M}\frac{N_{i}+1}{N_{i}-K_{i}+1}\left(\frac{m}{M}\right)\sum_{i=1}^{M}\frac{K_{i}}{N_{i}}\\ & = & \frac{k}{M}\sum_{i=1}^{M}\frac{\bar{N}+1}{\bar{N}-K_{i}+1}\left(\frac{m}{M\bar{N}}\right)\sum_{i=1}^{M}K_{i}\\ & = & \frac{k}{M}\sum_{i=1}^{M}\frac{\bar{N}+1}{\bar{N}-K_{i}+1}\left(\frac{m}{N}\right)K_{t}\\ & = & \left(\frac{mkK_{t}}{MN}\right)\sum_{i=1}^{M}\frac{\bar{N}+1}{\bar{N}-K_{i}+1}\\ & = & E_{SRS}\left(\nu_{r}\right) \end{array}$$

Since $E_{CTS}\left(\nu_{r}\right)=E_{SRS}\left(\nu_{r}\right)$, we focus on the ratio of E*_ATIS_* (*ν_r_*) over E*_CTS_*(*ν_r_*), which we call the relative expected observed rare units for ATIS and compute as

$$\begin{array}{c} {\text{RN}}_{ATIS}\left(\nu_{r}\right)=\frac{E_{ATIS}\left(\nu_{r}\right)}{E_{CTS}\left(\nu_{r}\right)}=\frac{N\sum_{i=1}^{M}\left(K_{i}\right)/\left(\bar{N}-K_{i}+1\right)}{K_{t}\sum_{i=1}^{M}\left(\bar{N}+1\right)/\left(\bar{N}-K_{i}+1\right)}. \end{array}$$

The relative expected number of observed rare units does not depend on *m* or *k*. Using Lagrange method, it can be shown that RN*_ATIS_*(*ν_r_*) is minimized when all *K*_*i*_ are equal to *K*_*t*_/*M* and its minimum would be *N*/(*N* + *M*). This will happen when the rare units are uniformly distributed over the population area, which implies that the population is not clustered.

The relative expected number of observed rare units for the buttercup population is 1.35, which means that ATIS will yield 35 percent more rare units, on average than SRS and CTS with the same final effective sample sizes. The RN*_ATIS_*(*ν_r_*) depends on the spatial distribution of the rare units over the study area. In Table 1, we compute RN*_ATIS_*(*ν_r_*) for 8 artificial populations with the same rarity as the buttercup population but with different spatial distributions of those 49 rare units over 12 PSUs. The highest value for RN*_ATIS_*(*ν_r_*) is 4.80 where all 49 rare units are located in two PSUs. The lowest is 1.00 where 4 rare units are located in 11 PSUs and 5 rare units is located in the last PSU. Theoretically, RN*_ATIS_*(*ν_r_*) can be as low as 300/312 = 0.962 but it cannot practically be smaller than 1 because it is impossible to allocate 49/12 = 4.08 rare units in each PSU.

**Table 1:** Eight imaginary populations are considered. Each population consists of 49 rare units. Those rare units are distributed among 12 PSUs which are resembling the buttercup population with different distribution of rare units. The numbers inside the table are the number of rare units in each PSU. The relative expected number of observed rare units, RN*_ATIS_*(*ν_r_*) are computed for each population.

Population	1	2	3	4	5	6	7	8
PSU								
1	25	20	20	20	20	10	5	4
2	24	19	19	19	10	10	5	4
3	0	10	5	2	10	10	5	4
4	0	0	5	2	9	10	5	4
5	0	0	0	2	0	9	5	4
6	0	0	0	2	0	0	5	4
7	0	0	0	2	0	0	5	4
8	0	0	0	0	0	0	5	4
9	0	0	0	0	0	0	5	4
10	0	0	0	0	0	0	4	4
11	0	0	0	0	0	0	0	4
12	0	0	0	0	0	0	0	5
RN*_ATIS_*(*ν_r_*)	4.8041	2.2735	2.2406	2.2274	1.9008	1.2823	1.0324	1.0001

### Simulation study

In the simulations, we distinguish between studies with the objective of estimating density (mean) and abundance (total) versus estimating occupancy (proportion). The written R codes for running the simulation are given in the S1 File.

#### Estimation of density and abundance

To study the efficiency of the estimators of ATIS, we simulated sampling of the buttercup population (Fig 1). The ATIS has similarities to the Gap-based inverse sampling (GIS) introduced by Panahbehagh and Brown [32]. However, SRS sampling and the conventional two-stage sampling outperform Gap-Based Inverse sampling (GIS) by a wide margin. Panahbehagh [11] reported that the SRS sample mean has smaller variance than GIS estimator. The GIS design is based on stratified sampling which is a special case of two-stage sampling where *m* = *M*. Using Table 1 of Panahbehagh and Brown [32] on page 9645, we computed the relative efficiency of Gap-based inverse sampling over the conventional two-stage (stratified) sampling and we found out that it ranges between 0.466 to 0.832 which means 53.4% to 16.8% loss of efficiency. Therefore we focused our simulation on the comparison between ATIS with the CTS and SRS designs.

We computed Relative Efficiency of the estimators over CTS as follows,

$$\begin{array}{c} \text{RE}\left(.\right)=\frac{\text{var}({\hat{\tau }}_{ts})}{\text{var}(.)}, \end{array}$$ (13)

where “.” stands for $\hat{\tau }$ in (5) or $\tilde{\tau }$ in (7) which we computed by Monte Carlo simulation method with 50,000 replications. But $\text{var}({\hat{\tau }}_{ts})$ is computed using its formula (e.g. Cochran, 1977) with equal sample size of $\bar{\nu }/m$ in each selected PSU, $\bar{\nu }$ is the mean of final sample size over those 50,000 replications of ATIS. The variance formula for conventional two-stage estimator is,

$$\begin{array}{c} \text{var}\left({\hat{\tau }}_{ts}\right)=\frac{M(M-m)}{m}\frac{1}{M-1}\sum_{i=1}^{M}{\left(\tau_{i}-\bar{\tau }\right)}^{2}+\frac{M}{m}\sum_{i=1}^{M}N_{i}\left(N_{i}-\frac{\bar{\nu }}{m}\right)\frac{m}{\bar{\nu }}\frac{\sum_{j=1}^{N_{i}}{\left(y_{ij}-\tau_{i}/N_{i}\right)}_{2}}{N_{i}-1}, \end{array}$$

where $\bar{\tau }=\left(1/M\right)\sum \tau_{i}$. We also computed the efficiencies, based on the efficiency definition by Särndal et al. [33], which is as follows,

$$\begin{array}{c} EF\left(.\right)=\frac{\text{var}({\hat{\tau }}_{SRS})}{\text{var}(.)}, \end{array}$$ (14)

where $\text{var}({\hat{\tau }}_{SRS})$ is again computed by its formula with size of $\bar{\nu }$. The simulation study was comprehensive for values of, *m*, *c* and *k*. The population was partitioned into *M* = 12 and *M* = 6 PSUs.

For the case of *M* = 12, *m* = 12, *k* = 2, 3, …, 10, and *c* = 0, 1, 2 the detailed results are presented in Table 2. For *c* = 0, the gain in relative efficiency for the Murthy’s estimator, $\hat{\tau }$, and the inadmissable estimator, $\tilde{\tau }$, are equivalent and ranges from 24% to 257%. The gain in efficiency ranges from 56% to 351%. The gains in relative efficiency increase as *k*_*i*_ increases.

**Table 2:** The variances of $\hat{\tau }$, $\tilde{\tau }$, ${\hat{\tau }}_{ts}$ and ${\hat{\tau }}_{SRS}$ are computed with the same effective sample sizes for the buttercups population when the population was partitioned into 12 PSUs. The relative efficiencies and the efficiencies of both estimators of ATIS are computed where *m* = 12.

*c*	*k*	$\text{var}(\tilde{\tau })$	$\text{var}(\tilde{\tau })$	$\text{var}({\hat{\tau }}_{ts})$	$\text{var}({\hat{\tau }}_{srs})$	$\text{RE}(\hat{\tau })$	$\text{EF}(\hat{\tau })$	$\text{RE}(\tilde{\tau })$	$\text{EF}(\tilde{\tau })$
0	2	7173.01	7173.01	8865.71	11186.62	1.24	1.56	1.24	1.56
	3	3944.57	3944.57	5569.68	7027.74	1.41	1.78	1.41	1.78
	4	2506.73	2506.73	3924.37	4951.71	1.57	1.98	1.57	1.98
	5	1730.62	1730.62	2937.95	3707.07	1.70	2.14	1.70	2.14
	6	1226.99	1226.99	2278.44	2874.90	1.86	2.34	1.86	2.34
	7	876.13	876.13	1807.45	2280.62	2.06	2.60	2.06	2.60
	8	617.47	617.47	1454.91	1835.79	2.36	2.97	2.36	2.97
	9	426.84	426.84	1180.89	1490.04	2.77	3.49	2.77	3.49
	10	269.05	269.05	961.19	1212.82	3.57	4.51	3.57	4.51
1	2	8170.04	8307.66	9723.35	12268.78	1.19	1.50	1.17	1.48
	3	4666.58	4708.53	6145.24	7753.97	1.32	1.66	1.31	1.65
	4	3078.24	3097.05	4356.06	5496.41	1.42	1.79	1.41	1.77
	5	2176.36	2191.40	3281.63	4140.72	1.51	1.90	1.50	1.89
	6	1600.59	1608.72	2566.13	3237.90	1.60	2.02	1.60	2.01
	7	1173.07	1181.01	2053.86	2591.53	1.75	2.21	1.74	2.19
	8	883.24	887.22	1670.80	2108.19	1.89	2.39	1.88	2.38
	9	664.54	668.13	1372.46	1731.75	2.07	2.61	2.05	2.59
	10	485.49	488.18	1133.74	1430.53	2.34	2.95	2.32	2.93
2	2	9549.18	10242.79	10636.93	13421.52	1.11	1.41	1.04	1.31
	3	5733.32	5950.00	6755.19	8523.60	1.18	1.49	1.14	1.43
	4	3876.63	3980.21	4810.68	6070.05	1.24	1.57	1.21	1.53
	5	2856.13	2924.76	3645.15	4599.39	1.28	1.61	1.25	1.57
	6	2152.17	2194.61	2869.08	3620.16	1.33	1.68	1.31	1.65
	7	1656.64	1685.76	2315.14	2921.21	1.40	1.76	1.37	1.73
	8	1328.43	1352.29	1898.54	2395.55	1.43	1.80	1.40	1.77
	9	1054.40	1071.50	1574.72	1986.96	1.49	1.88	1.47	1.85
	10	845.48	859.13	1316.13	1660.68	1.56	1.96	1.53	1.93

For *c* = 1, the gains in relative efficiency ranges from 19% to 134% for the Murthy’s estimator and from 17% to 132% for the inadmissible estimator. The efficiency gains for Murthy’s estimator is (50%, 195%) and for the inadmissable estimator is (48%, 193%). The differences between the gain in efficiency of Murthy’s estimator and those of the inadmissable estimators range from 0% to 2%. As in previous cases, the gains in efficiency increase as *k*_*i*_’s increase.

For *c* = 2, the gains in relative efficiency for the Murthy’s estimator ranges from 11% to 56% and for the inadmissible estimator ranges from 4% to 53%. The range of efficiency gains for the Murthy’s estimator is (41%, 96%) and for the inadmissible estimator is (31% to 93%). The range in the difference between efficiency gains for the Murthy’s estimator and the inadmissable estimator is from 2% to 7%. As in previous cases, the gains again increase as *k*_*i*_ increases.

As *c* increases, efficiency gains decrease ostensibly as a result of decreasing cluster sizes. We found that the inadmissable estimator is more efficient than SRS and CTS, RE > 1 and EF > 1 for all cases, and its gains are very close to Murthy’s estimator gains.

**Fig 2:**
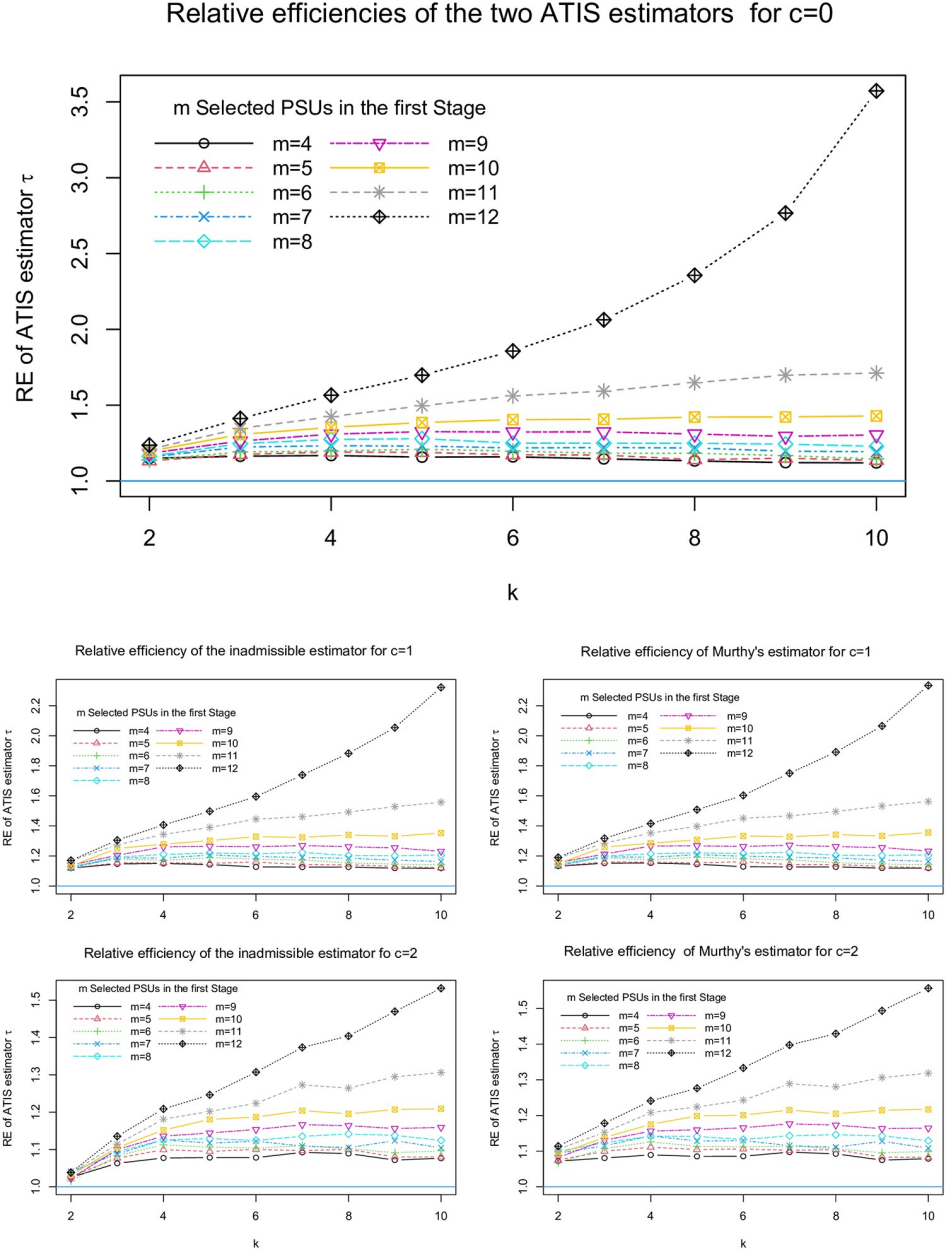
The buttercup population is partitioned into 12 PSUs of size 25 and the relative efficiency of Murthy’s and the inadmissable estimators of ATIS are computed for different values of *m*, *k* and *c* which are presented in 5 graphs.

In Fig 2 we present the relative efficiency for the population partitioned into 12 PSUs with equal size of 25. In this case, we ran the simulation for *m* = 4, 5, …, 12. The REs increase as *m*s increase. We found that the efficiency is much higher for *m* = 12 indicating the design performs better when the sampling design approaches the adaptive stratified inverse sampling design. Both estimators are more efficient than CTS in all cases. The behavior of the inadmissable estimator is very similar to the admissible estimator and there is little difference in RE. The REs generally increase as *k*_*i*_s increase but for smaller *m*s there are some cases that REs sightly decrease as *k*s increase. The REs decrease as *c* increase from 0 to 2.

**Fig 3:**
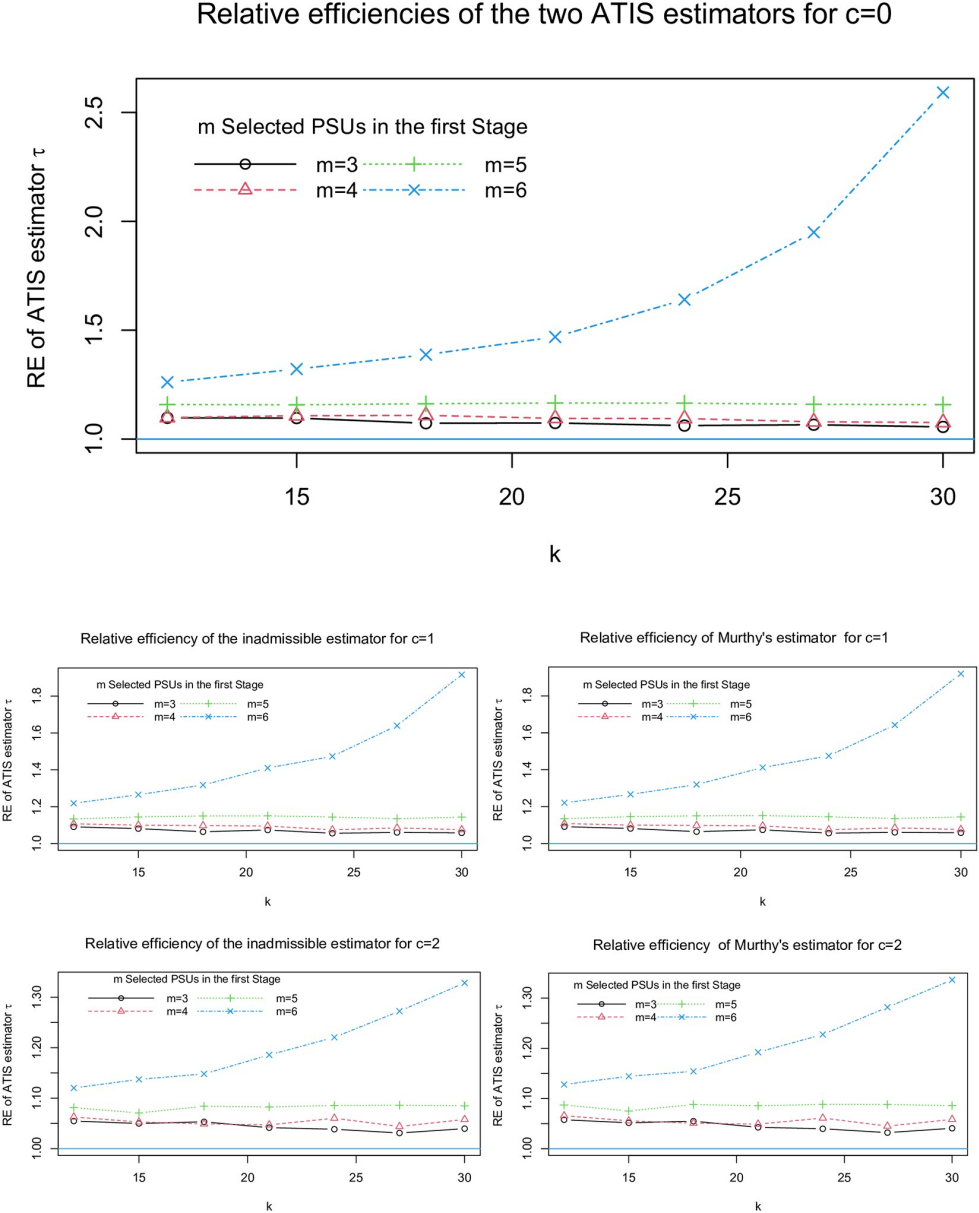
The buttercup population is partitioned into 6 PSUs of size 50 and the relative efficiency of Murthy’s and the inadmissable estimators of ATIS are computed for different values of *m*, *k* and *c* which are presented in 5 graphs.

To investigate the relationship between relative efficiency of ATIS and the size of PSUs, we partitioned the buttercup population into *M* = 6 PSUs of size 50 (Fig 3). We computed RE for *m* = 3, 4, 5, 6; *k* = 12, 15, 18, 21, 24, 27 and *c* = 0, 1, 2. The pattern in REs resembled the results for *M* = 12. Nevertheless, REs were higher for *N*_*i*_ = 25 than for *N*_*i*_. However, REs of both estimators exceeded 1 in all cases.

To understand the efficiency of ATIS in comparison to existing methods for rare and cluster sampling, we compared ATIS with TACS. We simulated sampling of the buttercup population (Fig 1) partitioned into 12 PSUs. Final sample sizes are random for TACS and ATIS so it is not possible to compare variances directly. Thus, we first compared each sampling design estimator with SRS with sample size equal to the effective final sample size of either ATIS or TACS by computing the estimator efficiency as (14).

Applying the Monte Carlo method, we computed the empirical variances of ATIS estimators for *m* = 9, 12; *c* = 0, 1, 2 and different *k*_*i*_s. Then, we computed the variance of a SRS of the same size as the effective final sample size of ATIS design corresponding to each case. Using the same approach, we computed the efficiency of Horvitz-Thompson estimator, ${\hat{\tau }}_{HT}$, and Hansen-Hurwitz estimator, ${\hat{\tau }}_{HH}$ for TACS for *m* = 9, 12; *c* = 0, 1, 2 and different *n*_*i*_s where *n*_*i*_ is the initial sample size from PSU *i*. For TACS details and notations see Seber and Salehi [1, 17].

We chose the closest effective final sample sizes of TACS, say E(*ν_TACS_*)’s, and of ATIS, say E(*ν_TACS_*)’s for given *m* and *c* with different *n*_*i*_ and *k*_*i*_ (Table 3). We found that for *m* = 12,${\hat{\tau }}_{HH}$ of TACS was the least efficient estimator. ATIS estimators were more efficient for moderate effective sample sizes whereas ${\hat{\tau }}_{HT}$, of TACS was more efficient for large effective final sample sizes. For example, when the effective final sample sizes were approximately larger than 90, ${\hat{\tau }}_{HT}$ of TACS became more efficient than ATIS estimators for *m* = 12. For *m* = 9, TACS estimators were more efficient for large and moderate effective final sample sizes and ATIS estimators were more efficient for smaller effective sample sizes. However, the relative efficiency for ATIS estimators were greater than one for all cases. But relative efficiency for TACS were less than 1 for some cases.

**Table 3:** The efficiencies of $\hat{\tau }$ and $\tilde{\tau }$ for ATIS and, those of ${\hat{\tau }}_{HT}$ and ${\hat{\tau }}_{HT}$ for TACS are computed for the buttercups population. They are computed for *m* = 9, 12 and *c* = 0, 1, 2. The initial sample sizes *k*_*i*_ and *n*_*i*_ are chosen in the way that we have closest E(*ν*_*TACS*_) and E(*ν*_*ATIS*_) in each row.

*m*	*c*	E(*ν*_*TACS*_)	$\text{EF}({\hat{\tau }}_{HT})$	$\text{EF}({\hat{\tau }}_{HH})$	E(*ν*_*ATIS*_)	$\text{EF}(\hat{\tau })$	$\text{EF}(\tilde{\tau })$
12	0	42.92	0.86	0.86	30.79	1.56	1.56
		69.78	1.54	0.99	61.56	1.98	1.98
		88.60	2.82	1.13	92.39	2.34	2.34
		103.48	5.10	1.25	107.81	2.60	2.60
		116.32	8.59	1.64	123.20	2.97	2.97
	1	35.28	0.85	0.89	28.33	1.50	1.48
		59.80	1.32	0.99	56.64	1.79	1.77
		77.86	2.12	1.10	70.81	1.90	1.89
		92.39	3.42	1.22	84.96	2.02	2.01
		104.97	5.38	1.32	99.15	2.21	2.21
	2	20.84	0.87	1.10	26.11	1.41	1.31
		39.06	1.01	1.14	39.15	1.49	1.43
		55.33	1.19	1.18	52.22	1.57	1.53
		70.13	1.40	1.23	65.29	1.61	1.57
		83.85	1.64	1.26	78.33	1.68	1.65
9	0	32.19	0.96	1.02	34.65	1.26	1.26
		52.31	1.43	1.14	57.72	1.33	1.33
		77.61	1.96	1.29	80.90	1.32	1.32
		87.24	1.98	1.32	92.41	1.31	1.31
		104.25	1.85	1.34	103.96	1.29	1.29
	1	26.46	0.95	1.02	21.23	1.16	1.14
		44.85	1.33	1.14	42.52	1.27	1.26
		58.40	1.67	1.23	53.12	1.27	1.26
		69.29	1.88	1.29	74.33	1.27	1.27
		87.33	1.96	1.34	84.99	1.26	1.26
	2	15.63	0.95	1.02	19.59	1.08	1.02
		29.30	1.10	1.17	29.37	1.13	1.10
		52.60	1.36	1.32	58.74	1.17	1.15
		90.71	1.59	1.35	88.12	1.16	1.16
		107.79	1.58	1.33	97.88	1.16	1.16

We also partitioned the buttercup population into 3 PSUs of size 100 and the simulation results are given in the S1 Fig.

#### Estimation of occupancy

The relative performance of adaptive cluster sampling and its different versions including TACS depends on neighborhood definition while ATIS is a neighborhood-free adaptive sampling design. On the other hand, when the within-network variance is small relative to the between-network variance, TACS performs poorly. In the extreme case the variable of interest is a binary, such as the presence or absence of an object a species within a sampling unit termed occupancy in the conservation literature [15, 19].

**Fig 4:**
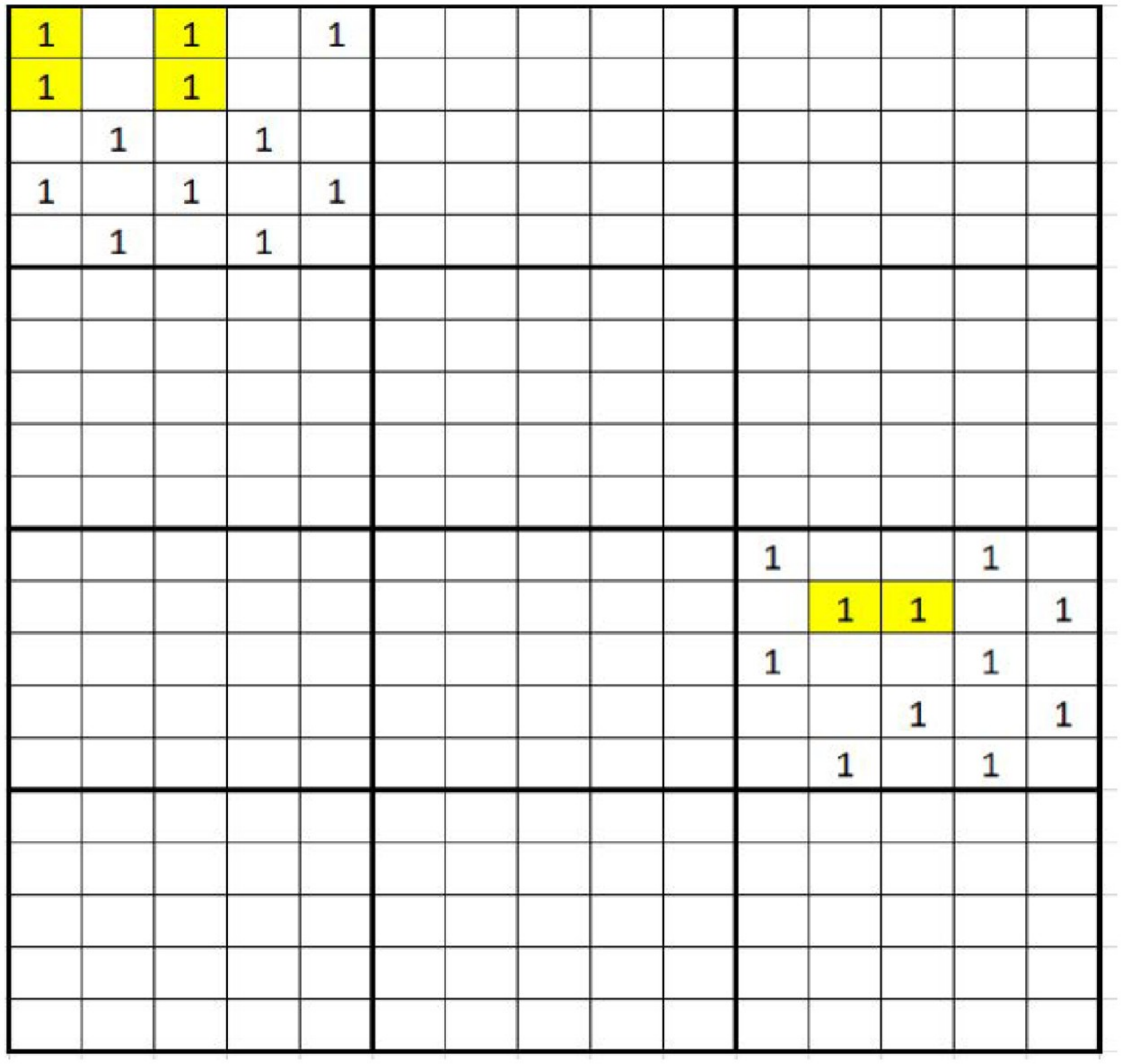
A population of 300 quadrats is partitioned into 12 PSUs of size 25. The variable of interest is binary. The value of those empty quadrats are 0. The value of each quadrat indicates the presence, 1, or the absence, 0, of a species. Two-stage adaptive cluster sampling is carried out and the neighborhood of a quadrat is the north, south, east and west quadrats. The highlighted quadrats are networks of size greater than one.

To investigate the performance of ATIS for estimating occupancy, we created a population using a binary variable with two large networks (Fig 4). If we use the usual neighborhood definition for which neighbors are the north, south, east and west, the two rare networks in the population will be broken into small networks. The relative efficiency (13) for ATIS estimators were computed where *c* = 0, *M* = 12 and *k*_*i*_ = 2, 3, …, 9, 10. The relative efficiency for TACS estimators were computed where *c* = 0, *M* = 12 and the initial sample, *n*_*i*_ = 2, 3, …, 9, 10. We found that the relative efficiency of TSAC estimators were less than 1, for all cases while those of ATIS estimators were substantially greater than 1 (Table 4). The reduced relative efficiency for TACS ranged from 16% to 23% for ${\hat{\tau }}_{HT}$ and from 29% to 49% for ${\hat{\tau }}_{HH}$. The gain in relative efficiency for ATIS ranged between 11% to 146%.

**Table 4:** The relative efficiencies of $\hat{\tau }$ and $\tilde{\tau }$ for ATIS and, those of ${\hat{\tau }}_{HT}$ and ${\hat{\tau }}_{HT}$ for TACS are computed for population of Fig 4, the presence and absence population. The relative efficiencies of ATIS and TACS are computed for *m* = 12, *c* = 0, with *k*_*i*_ = 2, 3, …, 10 for ATIS, and with *n*_*i*_ = 2, 3, …, 10 for TACS. For ATIS estimators, the relative efficiencies are calculated based on the effective final sample sizes of ATIS, $E(\nu_{ATIS})$. For TACS estimators, the relative efficiencies are calculated based on the effective final sample sizes of TACS, E(*ν_TACS_*).

*k*_*i*_; *n*_*i*_	E(*ν_TACS_*)	$\text{RE}({\hat{\tau }}_{HT})$	$\text{RE}({\hat{\tau }}_{HH})$	$E(\nu_{ATIS})$	$\text{RE}(\hat{\tau })$	$\text{RE}(\tilde{\tau })$
2	30.41	0.77	0.71	27.18	1.11	1.11
3	44.73	0.78	0.69	40.78	1.27	1.27
4	58.57	0.79	0.67	54.36	1.39	1.39
5	71.96	0.80	0.64	67.93	1.52	1.52
6	84.98	0.81	0.62	81.53	1.62	1.62
7	97.66	0.82	0.59	95.12	1.77	1.77
8	110.06	0.82	0.57	108.73	1.95	1.95
9	122.20	0.83	0.54	122.34	2.16	2.16
10	134.12	0.84	0.51	135.91	2.46	2.46

## Conclusion

The introduced ATIS design is a neighborhood-free and efficient adaptive sampling method that mimics how biologist would naturally search for a rare and clustered population. In addition, the design comes with an easy-to-compute estimator. The ATIS design yields significantly more rare units than its conventional counterparts. In comparison with TACS, ATIS is more efficient for small and moderate effective final sample sizes while TACS is more efficient for large effective final sample sizes. However, ATIS is considerably efficient when the variable of interest is binary whereas TACS performance is very poor for binary variable. Simulation studies indicate that ATIS is robustly efficient compared to TACS as there are cases that both estimators of TACS are less efficient than the conventional two-stage sampling even for rare and clustered populations.

When the population is rare and very clustered such that when a large cluster is located inside only one PSU, then there is a chance that all rare units will be missed unless all PSUs are not selected in the first stage. We therefore recommend implementing the stratified version of ATIS to whenever the budget and logistical constraints allow. We recommend choosing *k*_*i*_ proportional to size of the PSU. If *k*_*i*_ is too small the likelihood not sequentially sampling is high. The countervailing concern is that if *k*_*i*_’s are too large the budget and resources will be wasted in PSUs without rare units. Whenever the condition *C* is chosen such that the variable of interest for non-rare units are zero the easy-to-compute, inadmissable, estimator is as efficient as Murthy’s estimator. We therefore recommend choosing *C* such that the variable of interest for non-rare units is zero where possible.

## Supporting information

S1 Fig Simulation results. The graph presents the simulation study when the population is partitioned into 3 PSUs.
(TIF)

S1 File R codes. The R codes are used to run simulation studies.
(PDF)

S2 File (ZIP)

S1 AppendixDerivation of ${\hat{\tau }}_{i}$.(PDF)

S2 AppendixProof of $\hat{\text{var}}(\hat{\tau })=\hat{\text{var}}(\tilde{\tau })$ when *c* = 0.(PDF)
