# Supplementary figures and images for: Adaptive two-stage inverse sampling design to estimate density, abundance, and occupancy of rare and clustered populations
Source: PLoS One. 2021 Aug 18;16(8):e0255256. doi: 10.1371/journal.pone.0255256 (PMC8372892; doi:10.1371/journal.pone.0255256)

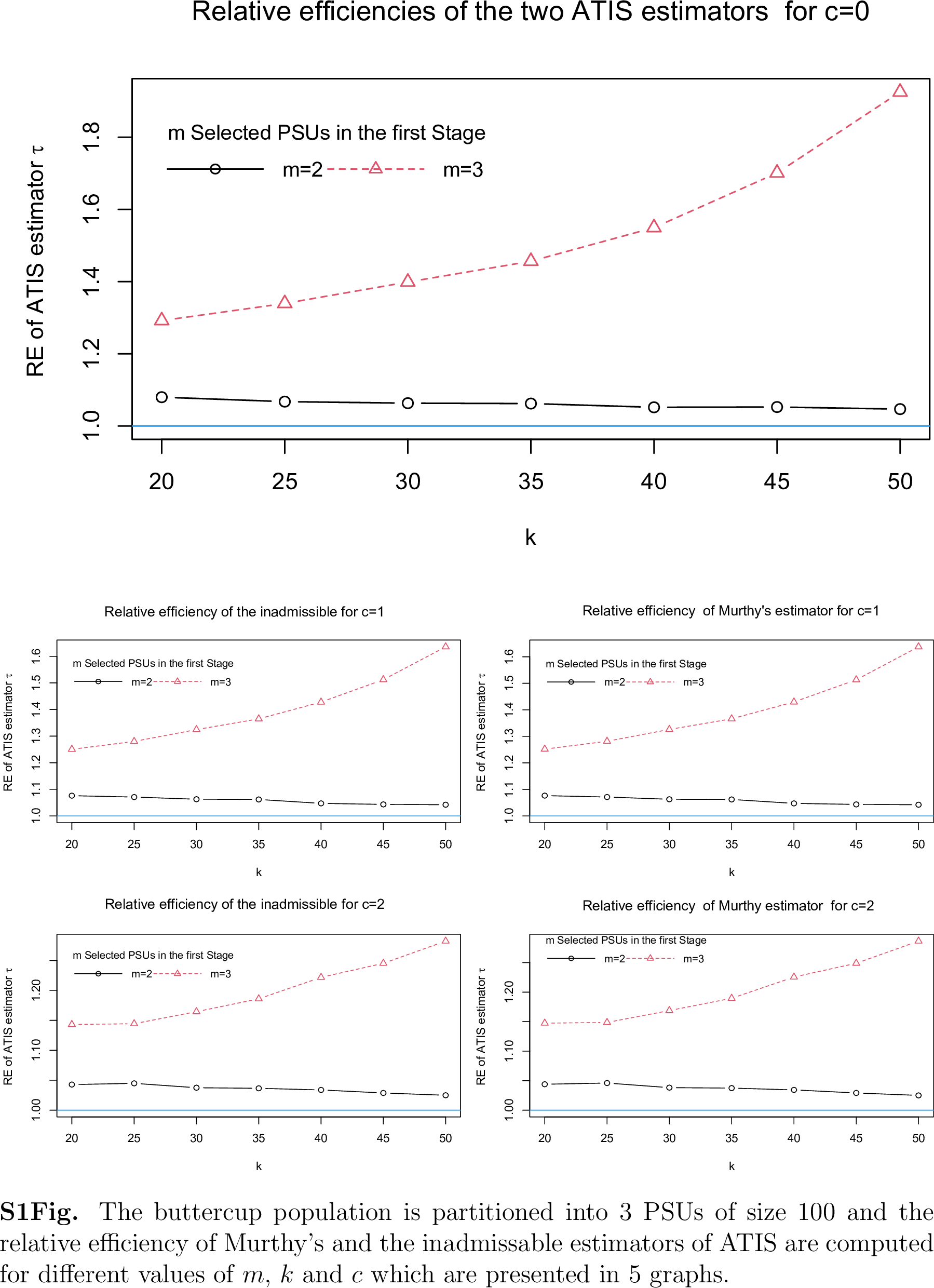

Supplement: S1 Fig — The graph presents the simulation study when the population is partitioned into 3 PSUs. (TIF) [file pone.0255256.s001.tif]
