# Supplementary material for: Adaptive two-stage inverse sampling design to estimate density, abundance, and occupancy of rare and clustered populations
Source: PLoS One. 2021 Aug 18;16(8):e0255256. doi: 10.1371/journal.pone.0255256 (PMC8372892; doi:10.1371/journal.pone.0255256)
Supplement: S1 File — The R codes are used to run simulation studies. (PDF) [file pone.0255256.s002.pdf]

S1File. R codes The R codes are used to run simulation studies.

Codes for simulating ATIS

```
invr=function(pop,k,c){
s=numeric(0)
N=length(pop)
po=1:N
s=sample(po,1)
sa=pop[s]
pop=pop[-s]
l=length(sa[sa<=c])
nu=1
while(l<k && nu<N ){
po=1:length(pop)
s=sample(po,1)
sa=c(sa,pop[s])
pop=pop[-s]
l=length(sa[sa<=c])
nu=length(sa)}
sa
}
tsin=function(P,m,k,c){
d=dim(P)
tau=0
tau1=0
n=0
n1=0
sam=sample(1:d[2],m)
Ps=P[,sam]
for(j in 1:m){
sa=invr(Ps[,j],k,c)
sa1=sa[sa<=c]
sa2=sa[sa>c]
nu=length(sa)
tau1=tau1+d[1]*mean(sa[-nu])
n=n+nu
mhat=d[1]*(k-1)/(nu-1)
n1=n1+length(sa2)
if(length(sa2)>0)
tau=tau+mhat*mean(sa1)+(d[1]-mhat)*mean(sa2)
else
tau=tau+mhat*mean(sa1)
}
tau1=d[2]*tau1/m
tau=d[2]*tau/m
```

```
re=c(n,tau,tau1,n1)
re}
```

```
tsiminv=function(P,m,k,c,r){
n2=0
re=tsin(P,m,k,c)
for(i in 1:(r-1))
re=rbind(re,tsin(P,m,k,c))
n=mean(re[,1])
tau=mean(re[,2])
tau1=mean(re[,3])
n1=mean(re[,4])
v=var(re[,2])
v1=var(re[,3])
d=dim(P)
N=d[1]*d[2]
ns=n/m
y=apply(P,2,sum)
w=as.vector(as.matrix(P))
for(j in 1:d[2])
n2=n2+ns*sum(P[,j]>c)/d[1]
n2=m/d[2]*n2
st=((d[2]/m*d[1]*(d[1]-ns)*sum(diag(var(P)))/ns)+d[2]*(d[2]-
m)*var(y)/m)
srs=N^2*((1-n/N)*var(w))/n
res=c(tau,"nu"=n,"#of rare"=n1,"#of
rareSt"=n2,"Var(tau)"=v,"Var(ybar-1)"=v1,
"var(st)"=st,"Var(srs)"=srs)
res}
tsiminv(but,12,7,0,50000)
```

Executions if ATIS

```
ren254t=numeric(0)
ren255t=numeric(0)
ren256t=numeric(0)
ren257t=numeric(0)
ren258t=numeric(0)
ren259t=numeric(0)
ren2510t=numeric(0)
ren2511t=numeric(0)
ren2512t=numeric(0)
for(i in 2:10){
ren254t=rbind(ren254t,tsiminv(but,4,i,2,50000))
ren255t=rbind(ren255t,tsiminv(but,5,i,2,50000))
ren256t=rbind(ren256t,tsiminv(but,6,i,2,50000))
ren257t=rbind(ren257t,tsiminv(but,7,i,2,50000))
ren258t=rbind(ren258t,tsiminv(but,8,i,2,50000))
ren259t=rbind(ren259t,tsiminv(but,9,i,2,50000))
ren2510t=rbind(ren2510t,tsiminv(but,10,i,2,50000))
ren2511t=rbind(ren2511t,tsiminv(but,11,i,2,50000))
ren2512t=rbind(ren2512t,tsiminv(but,12,i,2,50000))
```

```
}       2512,tsiminv(but,12,i,0,50000))
r
e
n
2
5
1
2
=
n
u
m
e
r
i
c
(
0
)
f
o
r
(
i
i
n
2
:
1
0
)
r
e
n
2
5
1
2
=
r
b
i
n
d
(
r
e
n
```

```
ren2511
ren254o=numeric(0)
ren255o=numeric(0)
ren256o=numeric(0)
ren257o=numeric(0)
ren258o=numeric(0)
ren259o=numeric(0)
ren2510o=numeric(0)
ren2511o=numeric(0)
for(i in 2:10){
ren254o=rbind(ren254o,tsiminv(but,4,i,1,50000))
ren255o=rbind(ren255o,tsiminv(but,5,i,1,50000))
ren256o=rbind(ren256o,tsiminv(but,6,i,1,50000))
ren257o=rbind(ren257o,tsiminv(but,7,i,1,50000))
ren258o=rbind(ren258o,tsiminv(but,8,i,1,50000))
ren259o=rbind(ren259o,tsiminv(but,9,i,1,50000))
ren2510o=rbind(ren25510o,tsiminv(but,10,i,1,50000))
ren2511o=rbind(ren2511o,tsiminv(but,11,i,1,50000))
}
ren2510o=numeric(0)
ren2512o=numeric(0)
for(i in 2:10)
ren2512o=rbind(ren2512o,tsiminv(but,12,i,1,50000))
ren2511o=rbind(ren2511o,tsiminv(but,11,i,1,50000))
for(i in 2:10){
ren254o=rbind(ren254o,tsiminv(but,4,i,1,50000))
ren255o=rbind(ren255o,tsiminv(but,5,i,1,50000))
ren256o=rbind(ren256o,tsiminv(but,6,i,1,50000))
ren257o=rbind(ren257o,tsiminv(but,7,i,1,50000))
ren258o=rbind(ren258o,tsiminv(but,8,i,1,50000))
ren259o=rbind(ren259o,tsiminv(but,9,i,1,50000))}
ren254t
ren255t
ren256t
ren257t
ren258t
ren259t
ren2510t
```

```
ren2511t
ren2512t
ren254t=numeric(0)
ren255t=numeric(0)
ren256t=numeric(0)
ren257t=numeric(0)
ren258t=numeric(0)
ren259t=numeric(0)
ren2510t=numeric(0)
ren2511t=numeric(0)
ren2512t=numeric(0)
for(i in 2:10){
ren254t=rbind(ren254t,tsiminv(but,4,i,2,50000))
ren255t=rbind(ren255t,tsiminv(but,5,i,2,50000))
ren256t=rbind(ren256t,tsiminv(but,6,i,2,50000))
ren257t=rbind(ren257t,tsiminv(but,7,i,2,50000))
ren258t=rbind(ren258t,tsiminv(but,8,i,2,50000))
ren259t=rbind(ren259t,tsiminv(but,9,i,2,50000))
ren2510t=rbind(ren2510t,tsiminv(but,10,i,2,50000))
ren2511t=rbind(ren2511t,tsiminv(but,11,i,2,50000))
ren2512t=rbind(ren2512t,tsiminv(but,12,i,2,50000))}
ren254o=numeric(0)
ren255o=numeric(0)
ren256o=numeric(0)
ren257o=numeric(0)
ren258o=numeric(0)
ren259o=numeric(0)
ren2510o=numeric(0)
ren2511o=numeric(0)
ren2512o=numeric(0)
for(i in 2:10){
ren254o=rbind(ren254o,tsiminv(but,4,i,1,50000))
ren255o=rbind(ren255o,tsiminv(but,5,i,1,50000))
ren256o=rbind(ren256o,tsiminv(but,6,i,1,50000))
ren257o=rbind(ren257o,tsiminv(but,7,i,1,50000))
ren258o=rbind(ren258o,tsiminv(but,8,i,1,50000))
ren259o=rbind(ren259o,tsiminv(but,9,i,1,50000))
ren2510o=rbind(ren2510o,tsiminv(but,10,i,1,50000))
ren2511o=rbind(ren2511o,tsiminv(but,11,i,1,50000))
ren2512o=rbind(ren2512o,tsiminv(but,12,i,1,50000))}
ren254=numeric(0)
ren255=numeric(0)
ren256=numeric(0)
ren257=numeric(0)
ren258=numeric(0)
```

```
ren259=numeric(0)
ren2510=numeric(0)
ren2511=numeric(0)
ren2512=numeric(0)
for(i in 7:10){
ren254=rbind(ren254,tsiminv(but,12,2,0,500))
ren255=rbind(ren255,tsiminv(but,5,i,0,50000))
ren256=rbind(ren256,tsiminv(but,6,i,0,50000))
ren257=rbind(ren257,tsiminv(but,7,i,0,50000))
ren258=rbind(ren258,tsiminv(but,8,i,0,50000))
ren259=rbind(ren259,tsiminv(but,9,i,0,50000))
ren2510=rbind(ren2510,tsiminv(but,10,i,0,50000))
ren2511=rbind(ren2511,tsiminv(but,11,i,0,50000))
ren2512=rbind(ren2512,tsiminv(but,12,i,0,50000))}
ren2512=rbind(ren2512,tsiminv(but,12,1,0,50000))
but50=as.matrix(but50)
tsiminv(but,12,1,0,50000)
sum(but)
ren504=numeric(0)
ren505=numeric(0)
ren506=numeric(0)
ren507=numeric(0)
ren508=numeric(0)
ren509=numeric(0)
ren5010=numeric(0)
ren5011=numeric(0)
ren5012=numeric(0)
for(i in 2:10){
ren504=rbind(ren504,tsiminv(but50,4,i,0,50000))
ren505=rbind(ren505,tsiminv(but50,5,i,0,50000))
ren506=rbind(ren506,tsiminv(but50,6,i,0,50000))
ren507=rbind(ren507,tsiminv(but50,7,i,0,50000))
ren508=rbind(ren508,tsiminv(but50,8,i,0,50000))
ren509=rbind(ren509,tsiminv(but50,9,i,0,50000))
ren5010=rbind(ren5010,tsiminv(but50,10,i,0,50000))
ren5011=rbind(ren5011,tsiminv(but50,11,i,0,50000))
ren5012=rbind(ren5012,tsiminv(but50,12,i,0,50000))}
ren504o=numeric(0)
ren505o=numeric(0)
ren506o=numeric(0)
ren507o=numeric(0)
ren508o=numeric(0)
ren509o=numeric(0)
ren5010o=numeric(0)
ren5011o=numeric(0)
ren5012o=numeric(0)
for(i in 2:10){
ren504o=rbind(ren504o,tsiminv(but50,4,i,1,50000))
```

```
ren505o=rbind(ren505o,tsiminv(but50,5,i,1,50000))
ren506o=rbind(ren506o,tsiminv(but50,6,i,1,50000))
ren507o=rbind(ren507o,tsiminv(but50,7,i,1,50000))
ren508o=rbind(ren508o,tsiminv(but50,8,i,1,50000))
ren509o=rbind(ren509o,tsiminv(but50,9,i,1,50000))
ren5010o=rbind(ren5010o,tsiminv(but50,10,i,1,50000))
ren5011o=rbind(ren5011o,tsiminv(but50,11,i,1,50000))
ren5012o=rbind(ren5012o,tsiminv(but50,12,i,1,50000))}
ren504t=numeric(0)
ren505t=numeric(0)
ren506t=numeric(0)
ren507t=numeric(0)
ren508t=numeric(0)
ren509t=numeric(0)
ren5010t=numeric(0)
ren5011t=numeric(0)
ren5012t=numeric(0)
for(i in 2:10){
ren504t=rbind(ren504t,tsiminv(but50,4,i,2,50000))
ren505t=rbind(ren505t,tsiminv(but50,5,i,2,50000))
ren506t=rbind(ren506t,tsiminv(but50,6,i,2,50000))
ren507t=rbind(ren507t,tsiminv(but50,7,i,2,50000))
ren508t=rbind(ren508t,tsiminv(but50,8,i,2,50000))
ren509t=rbind(ren509t,tsiminv(but50,9,i,2,50000))
ren5010t=rbind(ren5010t,tsiminv(but50,10,i,2,50000))
ren5011t=rbind(ren5011t,tsiminv(but50,11,i,2,50000))
ren5012t=rbind(ren5012t,tsiminv(but50,12,i,2,50000))}
ren503t
ren504t
ren505t
ren506t
for(i in 2:15){
ren503=rbind(ren503,tsiminv(but50,3,i,0,50000))
ren504=rbind(ren504,tsiminv(but50,4,i,0,50000))
ren505=rbind(ren505,tsiminv(but50,5,i,0,50000))
ren506=rbind(ren506,tsiminv(but50,6,i,0,50000))}
ren503o=numeric(0)
ren504o=numeric(0)
ren505o=numeric(0)
ren506o=numeric(0)
for(i in 2:15){
ren503o=rbind(ren503o,tsiminv(but50,3,i,1,50000))
ren504o=rbind(ren504o,tsiminv(but50,4,i,1,50000))
ren505o=rbind(ren505o,tsiminv(but50,5,i,1,50000))
ren506o=rbind(ren506o,tsiminv(but50,6,i,1,50000))}
```

```
ren503t= numeric(0)
ren504t=numeric(0)
ren505t=numeric(0)
ren506t=numeric(0)
for(i in 2:10){
ren503t=rbind(ren503t,tsiminv(but50,3,i,2,50000))
ren504t=rbind(ren504t,tsiminv(but50,4,i,2,50000))
ren505t=rbind(ren505t,tsiminv(but50,5,i,2,50000))
ren506t=rbind(ren506t,tsiminv(but50,6,i,2,50000))}
d=3*(4:10)
ren503=numeric(0)
ren504=numeric(0)
ren505=numeric(0)
ren506=numeric(0)
for(i in d){
ren503=rbind(ren503,tsiminv(but50,3,i,0,50000))
ren504=rbind(ren504,tsiminv(but50,4,i,0,50000))
ren505=rbind(ren505,tsiminv(but50,5,i,0,50000))
ren506=rbind(ren506,tsiminv(but50,6,i,0,50000))}
ren503o=numeric(0)
ren504o=numeric(0)
ren505o=numeric(0)
ren506o=numeric(0)
for(i in d){
ren503o=rbind(ren503o,tsiminv(but50,3,i,1,50000))
ren504o=rbind(ren504o,tsiminv(but50,4,i,1,50000))
ren505o=rbind(ren505o,tsiminv(but50,5,i,1,50000))
ren506o=rbind(ren506o,tsiminv(but50,6,i,1,50000))}
ren503t= numeric(0)
ren504t=numeric(0)
ren505t=numeric(0)
ren506t=numeric(0)
for(i in d){
ren503t=rbind(ren503t,tsiminv(but50,3,i,2,50000))
ren504t=rbind(ren504t,tsiminv(but50,4,i,2,50000))
ren505t=rbind(ren505t,tsiminv(but50,5,i,2,50000))
ren506t=rbind(ren506t,tsiminv(but50,6,i,2,50000))}
f=5*(4:10)
ren1002t
ren1003t
for(i in f){
ren1002=rbind(ren1002,tsiminv(but100,2,i,0,100000))
ren1003=rbind(ren1003,tsiminv(but100,3,i,0,100000))}
ren1002o=numeric(0)
ren1003o=numeric(0)
for(i in f){
```

```
ren1002o=rbind(ren1002o,tsiminv(but100,2,i,1,100000))
ren1003o=rbind(ren1003o,tsiminv(but100,3,i,1,100000))}
ren1002t=numeric(0)
ren1003t=numeric(0)
for(i in f){
ren1002t=rbind(ren1002t,tsiminv(but100,2,i,2,100000))
ren1003t=rbind(ren1003t,tsiminv(but100,3,i,2,100000))
}
but100=as.matrix(but100)
```

Execution for binary data

```
Arti=numeric(0)
for(i in 2:10)
Arti=rbind(Arti,tsiminv(hta,12,i,0,50000))
tsiminv(hta,12,3,0,500)
dim(hta)
dim(but)
dim(invr(P[,8],5,0))
tsin(P,12,6,0)
Indi=numeric(0)
for(i in 2:10)
Indi=rbind(Indi,tsiminv(Inb,12,i,0,50000))
write_xlsx(Indi,"c:/D/move/inv/Indi.xlsx")
Indi=data.frame(Indi)
```

Computation of TACS

```
pii=function(x,N,n)
p=1-choose(N-x,n)/choose(N,n)
varht=function(y,x,N,n){
p=pii(x,N,n)
nu=length(y)
v=sum(((1-p)/p)*y^2)
for (j in 1:(nu-1)) {
for (i in (j+1):nu) {
pij=p[i]+p[j]-(1-choose(N-x[i]-x[j],n)/choose(N,n))
v=v+2*(pij-p[i]*p[j])/(p[i]*p[j])*y[i]*y[j]
}}
return(v) }
tt=data.frame(nu,v,ef,nuI,vI,efI,nuII,vII,efII)
write_xlsx(ht9,"c:/D/move/inv/ht9.xlsx")
write_xlsx(Arti,"c:/D/move/inv/Indic.xlsx")
Arti=as.data.frame(Arti)
```
