# Supplementary material for: Adaptive two-stage inverse sampling design to estimate density, abundance, and occupancy of rare and clustered populations
Source: PLoS One. 2021 Aug 18;16(8):e0255256. doi: 10.1371/journal.pone.0255256 (PMC8372892; doi:10.1371/journal.pone.0255256)
Supplement: S2 File — (ZIP) [file pone.0255256.s003.zip › Codes for simulating ATIS.pdf]

Codes for simulating ATIS

```
invr=function(pop,k,c){
  s=numeric(0)
  N=length(pop)
  po=1:N
  s=sample(po,1)
  sa=pop[s]
  pop=pop[-s]
  l=length(sa[sa<=c])
  nu=1
  while(l<k && nu<N ){
    po=1:length(pop)
    s=sample(po,1)
    sa=c(sa,pop[s])
    pop=pop[-s]
    l=length(sa[sa<=c])
    nu=length(sa)}
  sa
}

tsin=function(P,m,k,c){
 d=dim(P)
 tau=0
 tau1=0
 n=0
 n1=0
 sam=sample(1:d[2],m)
 Ps=P[,sam]
 for(j in 1:m){
  sa=invr(Ps[,j],k,c)
  sa1=sa[sa<=c]
  sa2=sa[sa>c]
  nu=length(sa)
  tau1=tau1+d[1]*mean(sa[-nu])
  n=n+nu
  mhat=d[1]*(k-1)/(nu-1)
  n1=n1+length(sa2)
  if(length(sa2)>0)
   tau=tau+mhat*mean(sa1)+(d[1]-mhat)*mean(sa2)
  else
   tau=tau+mhat*mean(sa1)
 }
 tau1=d[2]*tau1/m
 tau=d[2]*tau/m
 re=c(n,tau,tau1,n1)
 re}
```

```
tsiminv=function(P,m,k,c,r){
 n2=0
 re=tsin(P,m,k,c)
 for(i in 1:(r-1))
  re=rbind(re,tsin(P,m,k,c))
 n=mean(re[,1])
 tau=mean(re[,2])
 tau1=mean(re[,3])
 n1=mean(re[,4])
 v=var(re[,2])
 v1=var(re[,3])
 d=dim(P)
 N=d[1]*d[2]
 ns=n/m
 y=apply(P,2,sum)
w=as.vector(as.matrix(P))
 for(j in 1:d[2])
  n2=n2+ns*sum(P[,j]>c)/d[1]
 n2=m/d[2]*n2
 st=((d[2]/m*d[1]*(d[1]-ns)*sum(diag(var(P)))/ns)+d[2]*(d[2]-m)*var(y)/m)
 srs=N^2*((1-n/N)*var(w))/n
               res=c(tau,"nu"=n,"#of       rare"=n1,"#of       rareSt"=n2,"Var(tau)"=v,"Var(ybar-1)"=v1,
  "var(st)"=st,"Var(srs)"=srs)
 res}

tsiminv(but,12,7,0,50000)
```

Executions if ATIS

```
ren254t=numeric(0)
ren255t=numeric(0)
ren256t=numeric(0)
ren257t=numeric(0)
ren258t=numeric(0)
ren259t=numeric(0)
ren2510t=numeric(0)
ren2511t=numeric(0)
ren2512t=numeric(0)
for(i in 2:10){
 ren254t=rbind(ren254t,tsiminv(but,4,i,2,50000))
            ren255t=rbind(ren255t,tsiminv(but,5,i,2,50000))
                        ren256t=rbind(ren256t,tsiminv(but,6,i,2,50000))
                                    ren257t=rbind(ren257t,tsiminv(but,7,i,2,50000))
                                                ren258t=rbind(ren258t,tsiminv(but,8,i,2,50000))
                                                ren259t=rbind(ren259t,tsiminv(but,9,i,2,50000))
                                                ren2510t=rbind(ren2510t,tsiminv(but,10,i,2,50000))
                                                ren2511t=rbind(ren2511t,tsiminv(but,11,i,2,50000))
                                                ren2512t=rbind(ren2512t,tsiminv(but,12,i,2,50000))
```

```
}
        ren2512=numeric(0)
        for(i in 2:10)
         ren2512=rbind(ren2512,tsiminv(but,12,i,0,50000))


         ren2511
        ren254o=numeric(0)
        ren255o=numeric(0)
        ren256o=numeric(0)
        ren257o=numeric(0)
        ren258o=numeric(0)
        ren259o=numeric(0)
        ren2510o=numeric(0)
        ren2511o=numeric(0)
        for(i in 2:10){
         ren254o=rbind(ren254o,tsiminv(but,4,i,1,50000))
         ren255o=rbind(ren255o,tsiminv(but,5,i,1,50000))
         ren256o=rbind(ren256o,tsiminv(but,6,i,1,50000))
         ren257o=rbind(ren257o,tsiminv(but,7,i,1,50000))
         ren258o=rbind(ren258o,tsiminv(but,8,i,1,50000))
         ren259o=rbind(ren259o,tsiminv(but,9,i,1,50000))
         ren2510o=rbind(ren25510o,tsiminv(but,10,i,1,50000))
         ren2511o=rbind(ren2511o,tsiminv(but,11,i,1,50000))
        }

        ren2510o=numeric(0)
        ren2512o=numeric(0)
        for(i in 2:10)
         ren2512o=rbind(ren2512o,tsiminv(but,12,i,1,50000))
         ren2511o=rbind(ren2511o,tsiminv(but,11,i,1,50000))


        for(i in 2:10){
         ren254o=rbind(ren254o,tsiminv(but,4,i,1,50000))
         ren255o=rbind(ren255o,tsiminv(but,5,i,1,50000))
         ren256o=rbind(ren256o,tsiminv(but,6,i,1,50000))
         ren257o=rbind(ren257o,tsiminv(but,7,i,1,50000))
         ren258o=rbind(ren258o,tsiminv(but,8,i,1,50000))
         ren259o=rbind(ren259o,tsiminv(but,9,i,1,50000))}

         ren254t
         ren255t
         ren256t
         ren257t
         ren258t
         ren259t
         ren2510t
```

```
ren2511t
ren2512t

ren254t=numeric(0)
ren255t=numeric(0)
ren256t=numeric(0)
ren257t=numeric(0)
ren258t=numeric(0)
ren259t=numeric(0)
ren2510t=numeric(0)
ren2511t=numeric(0)
ren2512t=numeric(0)
for(i in 2:10){
 ren254t=rbind(ren254t,tsiminv(but,4,i,2,50000))
 ren255t=rbind(ren255t,tsiminv(but,5,i,2,50000))
 ren256t=rbind(ren256t,tsiminv(but,6,i,2,50000))
 ren257t=rbind(ren257t,tsiminv(but,7,i,2,50000))
 ren258t=rbind(ren258t,tsiminv(but,8,i,2,50000))
 ren259t=rbind(ren259t,tsiminv(but,9,i,2,50000))
 ren2510t=rbind(ren2510t,tsiminv(but,10,i,2,50000))
 ren2511t=rbind(ren2511t,tsiminv(but,11,i,2,50000))
 ren2512t=rbind(ren2512t,tsiminv(but,12,i,2,50000))}

ren254o=numeric(0)
ren255o=numeric(0)
ren256o=numeric(0)
ren257o=numeric(0)
ren258o=numeric(0)
ren259o=numeric(0)
ren2510o=numeric(0)
ren2511o=numeric(0)
ren2512o=numeric(0)
for(i in 2:10){
 ren254o=rbind(ren254o,tsiminv(but,4,i,1,50000))
 ren255o=rbind(ren255o,tsiminv(but,5,i,1,50000))
 ren256o=rbind(ren256o,tsiminv(but,6,i,1,50000))
 ren257o=rbind(ren257o,tsiminv(but,7,i,1,50000))
 ren258o=rbind(ren258o,tsiminv(but,8,i,1,50000))
 ren259o=rbind(ren259o,tsiminv(but,9,i,1,50000))
 ren2510o=rbind(ren2510o,tsiminv(but,10,i,1,50000))
 ren2511o=rbind(ren2511o,tsiminv(but,11,i,1,50000))
 ren2512o=rbind(ren2512o,tsiminv(but,12,i,1,50000))}

ren254=numeric(0)
ren255=numeric(0)
ren256=numeric(0)
ren257=numeric(0)
ren258=numeric(0)
```

```
ren259=numeric(0)
ren2510=numeric(0)
ren2511=numeric(0)
ren2512=numeric(0)
for(i in 7:10){
 ren254=rbind(ren254,tsiminv(but,12,2,0,500))
 ren255=rbind(ren255,tsiminv(but,5,i,0,50000))
 ren256=rbind(ren256,tsiminv(but,6,i,0,50000))
 ren257=rbind(ren257,tsiminv(but,7,i,0,50000))
 ren258=rbind(ren258,tsiminv(but,8,i,0,50000))
 ren259=rbind(ren259,tsiminv(but,9,i,0,50000))
 ren2510=rbind(ren2510,tsiminv(but,10,i,0,50000))
 ren2511=rbind(ren2511,tsiminv(but,11,i,0,50000))
 ren2512=rbind(ren2512,tsiminv(but,12,i,0,50000))}
ren2512=rbind(ren2512,tsiminv(but,12,1,0,50000))
but50=as.matrix(but50)
tsiminv(but,12,1,0,50000)
sum(but)
ren504=numeric(0)
ren505=numeric(0)
ren506=numeric(0)
ren507=numeric(0)
ren508=numeric(0)
ren509=numeric(0)
ren5010=numeric(0)
ren5011=numeric(0)
ren5012=numeric(0)
for(i in 2:10){
 ren504=rbind(ren504,tsiminv(but50,4,i,0,50000))
 ren505=rbind(ren505,tsiminv(but50,5,i,0,50000))
 ren506=rbind(ren506,tsiminv(but50,6,i,0,50000))
 ren507=rbind(ren507,tsiminv(but50,7,i,0,50000))
 ren508=rbind(ren508,tsiminv(but50,8,i,0,50000))
 ren509=rbind(ren509,tsiminv(but50,9,i,0,50000))
 ren5010=rbind(ren5010,tsiminv(but50,10,i,0,50000))
 ren5011=rbind(ren5011,tsiminv(but50,11,i,0,50000))
 ren5012=rbind(ren5012,tsiminv(but50,12,i,0,50000))}
ren504o=numeric(0)
ren505o=numeric(0)
ren506o=numeric(0)
ren507o=numeric(0)
ren508o=numeric(0)
ren509o=numeric(0)
ren5010o=numeric(0)
ren5011o=numeric(0)
ren5012o=numeric(0)
for(i in 2:10){
 ren504o=rbind(ren504o,tsiminv(but50,4,i,1,50000))
```

```
 ren505o=rbind(ren505o,tsiminv(but50,5,i,1,50000))
 ren506o=rbind(ren506o,tsiminv(but50,6,i,1,50000))
 ren507o=rbind(ren507o,tsiminv(but50,7,i,1,50000))
 ren508o=rbind(ren508o,tsiminv(but50,8,i,1,50000))
 ren509o=rbind(ren509o,tsiminv(but50,9,i,1,50000))
 ren5010o=rbind(ren5010o,tsiminv(but50,10,i,1,50000))
 ren5011o=rbind(ren5011o,tsiminv(but50,11,i,1,50000))
 ren5012o=rbind(ren5012o,tsiminv(but50,12,i,1,50000))}

ren504t=numeric(0)
ren505t=numeric(0)
ren506t=numeric(0)
ren507t=numeric(0)
ren508t=numeric(0)
ren509t=numeric(0)
ren5010t=numeric(0)
ren5011t=numeric(0)
ren5012t=numeric(0)
for(i in 2:10){
 ren504t=rbind(ren504t,tsiminv(but50,4,i,2,50000))
 ren505t=rbind(ren505t,tsiminv(but50,5,i,2,50000))
 ren506t=rbind(ren506t,tsiminv(but50,6,i,2,50000))
 ren507t=rbind(ren507t,tsiminv(but50,7,i,2,50000))
 ren508t=rbind(ren508t,tsiminv(but50,8,i,2,50000))
 ren509t=rbind(ren509t,tsiminv(but50,9,i,2,50000))
 ren5010t=rbind(ren5010t,tsiminv(but50,10,i,2,50000))
 ren5011t=rbind(ren5011t,tsiminv(but50,11,i,2,50000))
 ren5012t=rbind(ren5012t,tsiminv(but50,12,i,2,50000))}


ren503t
ren504t
ren505t
ren506t
for(i in 2:15){
 ren503=rbind(ren503,tsiminv(but50,3,i,0,50000))
 ren504=rbind(ren504,tsiminv(but50,4,i,0,50000))
 ren505=rbind(ren505,tsiminv(but50,5,i,0,50000))
 ren506=rbind(ren506,tsiminv(but50,6,i,0,50000))}
ren503o=numeric(0)
ren504o=numeric(0)
ren505o=numeric(0)
ren506o=numeric(0)
for(i in 2:15){
 ren503o=rbind(ren503o,tsiminv(but50,3,i,1,50000))
 ren504o=rbind(ren504o,tsiminv(but50,4,i,1,50000))
 ren505o=rbind(ren505o,tsiminv(but50,5,i,1,50000))
 ren506o=rbind(ren506o,tsiminv(but50,6,i,1,50000))}
```

```
ren503t= numeric(0)
ren504t=numeric(0)
ren505t=numeric(0)
ren506t=numeric(0)
for(i in 2:10){
 ren503t=rbind(ren503t,tsiminv(but50,3,i,2,50000))
 ren504t=rbind(ren504t,tsiminv(but50,4,i,2,50000))
 ren505t=rbind(ren505t,tsiminv(but50,5,i,2,50000))
 ren506t=rbind(ren506t,tsiminv(but50,6,i,2,50000))}

d=3*(4:10)
ren503=numeric(0)
ren504=numeric(0)
ren505=numeric(0)
ren506=numeric(0)
for(i in d){
 ren503=rbind(ren503,tsiminv(but50,3,i,0,50000))
 ren504=rbind(ren504,tsiminv(but50,4,i,0,50000))
 ren505=rbind(ren505,tsiminv(but50,5,i,0,50000))
 ren506=rbind(ren506,tsiminv(but50,6,i,0,50000))}
ren503o=numeric(0)
ren504o=numeric(0)
ren505o=numeric(0)
ren506o=numeric(0)
for(i in d){
 ren503o=rbind(ren503o,tsiminv(but50,3,i,1,50000))
 ren504o=rbind(ren504o,tsiminv(but50,4,i,1,50000))
 ren505o=rbind(ren505o,tsiminv(but50,5,i,1,50000))
 ren506o=rbind(ren506o,tsiminv(but50,6,i,1,50000))}
ren503t= numeric(0)
ren504t=numeric(0)
ren505t=numeric(0)
ren506t=numeric(0)
for(i in d){
 ren503t=rbind(ren503t,tsiminv(but50,3,i,2,50000))
 ren504t=rbind(ren504t,tsiminv(but50,4,i,2,50000))
 ren505t=rbind(ren505t,tsiminv(but50,5,i,2,50000))
 ren506t=rbind(ren506t,tsiminv(but50,6,i,2,50000))}

f=5*(4:10)
ren1002t
ren1003t
for(i in f){
 ren1002=rbind(ren1002,tsiminv(but100,2,i,0,100000))
 ren1003=rbind(ren1003,tsiminv(but100,3,i,0,100000))}
ren1002o=numeric(0)
ren1003o=numeric(0)
for(i in f){
```

```
 ren1002o=rbind(ren1002o,tsiminv(but100,2,i,1,100000))
 ren1003o=rbind(ren1003o,tsiminv(but100,3,i,1,100000))}

ren1002t=numeric(0)
ren1003t=numeric(0)
for(i in f){
 ren1002t=rbind(ren1002t,tsiminv(but100,2,i,2,100000))
 ren1003t=rbind(ren1003t,tsiminv(but100,3,i,2,100000))
}

but100=as.matrix(but100)
```

Execution for binary data

```
 Arti=numeric(0)
 for(i in 2:10)
   Arti=rbind(Arti,tsiminv(hta,12,i,0,50000))

 tsiminv(hta,12,3,0,500)
 dim(hta)
dim(but)
dim(invr(P[,8],5,0))
tsin(P,12,6,0)
Indi=numeric(0)
for(i in 2:10)
 Indi=rbind(Indi,tsiminv(Inb,12,i,0,50000))
write_xlsx(Indi,"c:/D/move/inv/Indi.xlsx")
Indi=data.frame(Indi)
```

Computation of TACS

```
pii=function(x,N,n)
 p=1-choose(N-x,n)/choose(N,n)
varht=function(y,x,N,n){
 p=pii(x,N,n)
 nu=length(y)
 v=sum(((1-p)/p)*y^2)
 for (j in 1:(nu-1)) {
  for (i in (j+1):nu) {
   pij=p[i]+p[j]-(1-choose(N-x[i]-x[j],n)/choose(N,n))
   v=v+2*(pij-p[i]*p[j])/(p[i]*p[j])*y[i]*y[j]
   }}
return(v) }

tt=data.frame(nu,v,ef,nuI,vI,efI,nuII,vII,efII)
write_xlsx(ht9,"c:/D/move/inv/ht9.xlsx")
write_xlsx(Arti,"c:/D/move/inv/Indic.xlsx")
Arti=as.data.frame(Arti)
```
