# Supplementary material for: Adaptive two-stage inverse sampling design to estimate density, abundance, and occupancy of rare and clustered populations
Source: PLoS One. 2021 Aug 18;16(8):e0255256. doi: 10.1371/journal.pone.0255256 (PMC8372892; doi:10.1371/journal.pone.0255256)
Supplement: S2 File — (ZIP) [file pone.0255256.s003.zip › SF1.pdf]

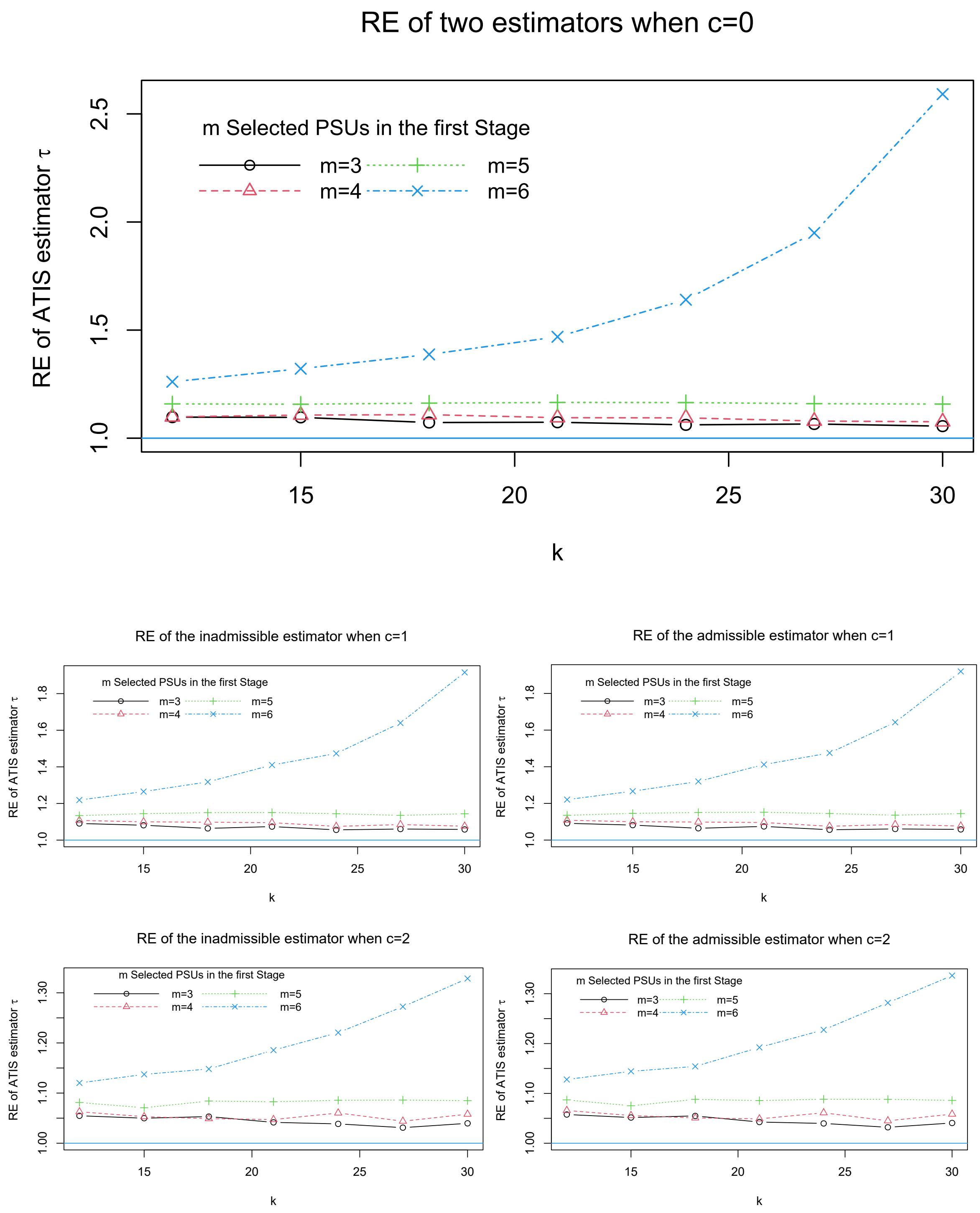


Figure 3: The buttercup population is partitioned into 6 PSUs of size 50 and the relative efficiency of admissible and inadmissable estimators of ATIS are computed for different values of $m$, $k$ and $c$ which are presented in 5 graphs.
