# Supplementary material for: Adaptive two-stage inverse sampling design to estimate density, abundance, and occupancy of rare and clustered populations
Source: PLoS One. 2021 Aug 18;16(8):e0255256. doi: 10.1371/journal.pone.0255256 (PMC8372892; doi:10.1371/journal.pone.0255256)
Supplement: S1 Appendix — (PDF) [file pone.0255256.s004.pdf]

S1 Appendix: Derivation of $\hat{\tau}_i$.

If $\nu_i > k_i$, i.e. the one at a time sampling part is carried out in PSU i. To evaluate $P(j|s_i)$ in (1), let D_{ij} be the number of ways that the selected sample s_i can be constructed such that unit j in PSU i is the first selected unit, and let D_i be the number of ways that the selected sample s_i can be constructed. Thus, $P(j|s_i) = D_{ij}/D_i$. If unit j belongs to $\mathcal{S}_{iC'}$, one of the $k_i - 1$ remaining units in s_i should be allocated as the last selected unit so that $D_{ij} = (k_i - 1)(\nu_i - 2)!$ and $D_i = k_i(\nu_i - 1)!$. We therefore have $P(j|s_i) = D_{ij}/D_i = (k_i - 1)/(k_i(\nu_i - 1))$ for $j \in \mathcal{S}_{iC'}$. If unit j belongs to $\mathcal{S}_{iC}$, one of the k_i units in s_i should be allocated as the last selected unit so that $D_{ij} = k_i(\nu_i - 2)!$ and the same as before $D_i = k_i(\nu_i - 1)!$. We therefore have $P(j|s_i) = D_{ij}/D_i = 1/(\nu_i - 1)$ for $j \in \mathcal{S}_{iC}$. We also have $p_{i,j} = 1/N_i$ for all j and i. Substituting the probabilities into (1), we have

$$\begin{aligned}
\hat{\tau}_i &= \sum_{j \in s_i} \frac{P(j|s_i)}{p_{j,i}} y_{ij} = N_i \sum_{j \in s_i} P(j|s_i) y_{ij} \\
&= N_i \left(\frac{1}{k_i} \sum_{j \in \mathcal{S}_{iC'}} \frac{k_i - 1}{\nu_i - 1} y_{ij} + \frac{1}{\nu_i - k_i} \sum_{j \in \mathcal{S}_{iC}} \frac{\nu_i - k_i}{\nu_i - 1} y_{ij} \right) \\
&= N_i \left(\hat{P}_i \overline{y}_{iC'} + (1 - \hat{P}_i) \overline{y}_{iC} \right)
\end{aligned}$$

If $k_i = \nu_i$, i.e. the one at a time part of sampling is not carried out in PSU i, so that $\hat{P}_i$ will be 1 and $\hat{\tau}_i = N_i \overline{y}_{iC'}$ which is given by (3).
