# Supplementary material for: Adaptive two-stage inverse sampling design to estimate density, abundance, and occupancy of rare and clustered populations
Source: PLoS One. 2021 Aug 18;16(8):e0255256. doi: 10.1371/journal.pone.0255256 (PMC8372892; doi:10.1371/journal.pone.0255256)
Supplement: S2 Appendix — (PDF) [file pone.0255256.s005.pdf]

**S2 Appendix: Proof of $\widehat{\text{var}}(\hat{\tau}) = \widehat{\text{var}}(\tilde{\tau})$ when $c = 0$.**

Since $y_{ij} = 0$ for all non-rare units $\bar{y}_{\nu_i - 1} = (\nu_i - k_i)/(\nu_i - 1)\bar{y}_{iC}$. Using simple algebra, we can show that

$$\sum_{j=1}^{\nu_i - 1}(y_{ij} - \bar{y}_{\nu_i - 1})^2 = \sum_{j \in S_{iC}}(y_{ij} - \frac{\nu_i - k_i}{\nu_i - 1}\bar{y}_{iC})^2 + (k_i - 1)\left(\frac{\nu_i - k_i}{\nu_i - 1}\right)^2 \bar{y}_{iC}^2$$

Adding and subtracting $\bar{y}_{iC}$ inside of the bracket of the first term on the right-hand side, we will have,

$$\begin{aligned}\sum_{j=1}^{\nu_i - 1}(y_{ij} - \bar{y}_{\nu_i - 1})^2 &= \sum_{j \in S_{iC}}(y_{ij} - \bar{y}_{iC})^2 + (\nu_i - k_i)\left(\frac{k_i - 1}{\nu_i - 1}\right)^2 \bar{y}_{iC}^2 + (k_i - 1)\left(\frac{\nu_i - k_i}{\nu_i - 1}\right)^2 \bar{y}_{iC}^2 \\ &= \sum_{j \in S_{iC}}(y_{ij} - \bar{y}_{iC})^2 + \frac{(k_i - 1)(\nu_i - k_i)}{\nu_i - 1}\bar{y}_{iC}^2\end{aligned}$$

Multiplying both sides by $N_i^2(1 - (\nu_i - 1)/((\nu_i - 1)(\nu_i - 2))$, the left-hand side will be $\widehat{\text{var}}(\tilde{\tau})$ and we have,

$$\begin{aligned}\widehat{\text{var}}(\tilde{\tau}) &= \frac{N_i(N_i - \nu_i + 1)}{(\nu_i - 1)(\nu_i - 2)}\sum_{j \in S_{iC}}(y_{ij} - \bar{y}_{iC})^2 + \frac{N_i(N_i - \nu_i + 1)(k_i - 1)(\nu_i - k_i)}{(\nu_i - 1)^2(\nu_i - 2)}\bar{y}_{iC}^2 \\ &= Bs_{iC}^2 + \widehat{\text{var}}(\hat{P})y_{iC}^2\end{aligned}$$

where $B$ is defined in (4). Since $y_{iC'}^2 = 0$ and $s_{iC'}^2 = 0$, the right-hand side is $\widehat{\text{var}}(\hat{\tau})$.
